# *ERF5.1* modulates carotenoid accumulation by interacting with *CCD4.1 in Lycium*

**DOI:** 10.1093/hr/uhad230

**Published:** 2023-11-17

**Authors:** Jianhua Zhao, Yuhui Xu, Haoxia Li, Xinlei Zhu, Yue Yin, Xiyan Zhang, Xiaoya Qin, Jun Zhou, Linyuan Duan, Xiaojie Liang, Ting Huang, Bo Zhang, Ru Wan, Zhigang Shi, Youlong Cao, Wei An

**Affiliations:** National Wolfberry Engineering Research Center/Wolfberry Science Research Institute, Ningxia Academy of Agriculture and Forestry Sciences, Yinchuan, 750002, China; National Wolfberry Engineering Research Center/Wolfberry Science Research Institute, Ningxia Academy of Agriculture and Forestry Sciences, Yinchuan, 750002, China; Institute of Forestry and Grassland Ecology, Ningxia Academy of Agriculture and Forestry Sciences, Yinchuan, 750002, China; National Wolfberry Engineering Research Center/Wolfberry Science Research Institute, Ningxia Academy of Agriculture and Forestry Sciences, Yinchuan, 750002, China; National Wolfberry Engineering Research Center/Wolfberry Science Research Institute, Ningxia Academy of Agriculture and Forestry Sciences, Yinchuan, 750002, China; National Wolfberry Engineering Research Center/Wolfberry Science Research Institute, Ningxia Academy of Agriculture and Forestry Sciences, Yinchuan, 750002, China; College of Biological Science & Engineering, North Minzu University, Yinchuan 750021, China; National Wolfberry Engineering Research Center/Wolfberry Science Research Institute, Ningxia Academy of Agriculture and Forestry Sciences, Yinchuan, 750002, China; National Wolfberry Engineering Research Center/Wolfberry Science Research Institute, Ningxia Academy of Agriculture and Forestry Sciences, Yinchuan, 750002, China; National Wolfberry Engineering Research Center/Wolfberry Science Research Institute, Ningxia Academy of Agriculture and Forestry Sciences, Yinchuan, 750002, China; National Wolfberry Engineering Research Center/Wolfberry Science Research Institute, Ningxia Academy of Agriculture and Forestry Sciences, Yinchuan, 750002, China; National Wolfberry Engineering Research Center/Wolfberry Science Research Institute, Ningxia Academy of Agriculture and Forestry Sciences, Yinchuan, 750002, China; National Wolfberry Engineering Research Center/Wolfberry Science Research Institute, Ningxia Academy of Agriculture and Forestry Sciences, Yinchuan, 750002, China; National Wolfberry Engineering Research Center/Wolfberry Science Research Institute, Ningxia Academy of Agriculture and Forestry Sciences, Yinchuan, 750002, China; National Wolfberry Engineering Research Center/Wolfberry Science Research Institute, Ningxia Academy of Agriculture and Forestry Sciences, Yinchuan, 750002, China

## Abstract

Carotenoids are important natural pigments and have medical and health functions for humans. Carotenoid cleavage dioxygenase 4 (*CCD4*) and ethylene responsive factor (ERF) participate in carotenoid metabolism, but their roles in *Lycium* have not been discovered. Here, we annotated *LbCCD*s from the *Lycium* reference genome and found that *LbCCD4.1* expression was significantly correlated with the carotenoid metabolites during *Lycium* five fruit developmental stages. Over-expression of *LbCCD4.1* in NQ’s leaves resulted in a series of significantly lower contents of carotenoid metabolites, including β-carotene and β-cryptoxanthin. Moreover, *LbERF5.1*, a transcription factor belonging to the ERF family that was located in the nucleus, was isolated. Significant reductions in the carotenoids, especially lutein, violaxanthin and their derivatives, were observed in over-expressing *ERF5.1* transgenic NQ’s leaves. Over-expression or virus-induced gene silencing of *LbERF5.1* in NQ’s leaves induced a consistent up- or down-expression, respectively, of *LbCCD4.1*. Furthermore, yeast one-hybrid and dual-luciferase reporter assays showed that *ERF5.1* interacted with the promoter of *CCD4.1* to increase its expression, and *LbERF5.1* could bind to any one of the three predicted binding sites in the promoter of *LbCCD4.1*. A transcriptome analysis of *LbERF5.1* and *LbCCD4.1* over-expressed lines showed similar global transcript expression, and geranylgeranyl diphosphate synthase, phytoene synthase, lycopene δ-cyclase cytochrome, cytochrome P450-type monooxygenase 97A, cytochrome P450-type monooxygenase 97C, and zeaxanthin epoxidase in the carotenoid biosynthesis pathway were differentially expressed. In summary, we uncovered a novel molecular mechanism of carotenoid accumulation that involved an interaction between *ERF5.1* and *CCD4.1*, which may be used to enhance carotenoid in *Lycium*.

## Introduction

Wolfberry (*Lycium barbarum* Linn.) is a traditional Chinese herbal medicine having a bright red color and soft pulp fruit, which contains rich bioactive ingredients, including carotenoids [1]. Specifically, goji (the dried *Lycium* fruit) contains monohydroxylutein and dihydroxylutein, α-carotene, β-carotene, β-cryptoxanthin, and zeaxanthin; and the carotenoid fatty acid esters are mainly zeaxanthin dipalmitate, zeaxanthin monopalmitate, and β-cryptoxanthin palmitate, as well as other metabolites [2–5]. During the fruit development and ripening of *Lycium*, carotenoids gradually accumulate, beginning with the discoloration stage, and the content can reach up to 400 μg/g (fresh fruit), indicating that the fruit is a potentially important source of carotenoids [6]. The carotenoid content in fruit is diverse among different accessions [7] and the metabolite diversity can be driven by environmental factors [8]. In addition, the leaf of wolfberry is also an organ for storing carotenoid [9, 10].

Carotenoids are important natural pigments widely distributed in plants, algae, fungi and a few animals. They are the source of gorgeous colors, like yellow, orange and red, in plants, fruits and flowers [11]. To date, over 750 natural carotenoids have been discovered [12]. In the food industry, carotene can be used as an additive for food coloring and nutritional fortification. In the cosmetics industry, carotene is mainly added to lipstick and rouge. In the pharmaceutical industry, carotene is used owing to its physiological functions of stimulating immunity and preventing metastasis and cardiovascular diseases [13–15]. It can also be used to treat diseases caused by vitamin A deficiency. In addition, carotenoids have important functions in plants, like responding to environmental stimuli and enhancing salt and drought-stress tolerance levels by boosting oxidative resistance, as seen in Arabidopsis and tobacco [16, 17].

Over the past decade, significant progress has been made in carotenoid biosynthesis pathway in plants [18, 19], including several enzyme catalysis steps. However, the carotenoid degradation pathway is more complicated than the biosynthesis pathway [20]. In plants, the enzymes involved in carotenoid degradation are termed carotenoid cleavage dioxygenases (CCDs). At present, 13 CCD family members, including six CCD subfamilies (*CCD1*, *−2*, *−4*, *−7*, *−8*, and *−10*) and seven subfamilies of NCED (*NCED1–6* and *NCED9*), have been found in plants [21, 22]. Many CCD enzymes can cleave the conjugated C-C double bonds in carotenoids to produce different apocarotenoids [21]. *CCD1* and *CCD2* are responsible for carotenoid degradation and the depletion of the carotenoid pools in saffron and spring crocuses [23, 24]. In addition to *CCD1* and *CCD2*, *CCD4* is the most reported subfamily involved in carotenoid degradation. In the herb chrysanthemum, *CmCCD4a* contributes to carotenoid degradation, resulting in a white color [25]. In peach fruit, the sequence and expression associations between *PpCCD4* and flesh color, carotenoid metabolites phenotype have been observed, indicating *PpCCD4*’s function in flesh color formation [26, 27]. The genome-wide identification of CCDs in honeysuckle reveals that *LjCCD4* and *LjCCD1b* are highly expressed in petals. Expressed *LjCCD4* and *LjCCD1b* proteins can convert β-carotene, lutein and 10′-apo-β-carotene into colorless and volatile substances, resulting in color changes [28]. In carrot, *DcCCD4* affects the accumulation of carotenoids through the cleavage of α-carotene and β-carotene in carrot taproots [29]. Genome-wide association studies (GWAS) reveal that ZEP and CCD4 are responsible for seed carotenoid degradation, and ZEP is an upstream contributor to carotenoid degradation in *Arabidopsis* seeds [30, 31]. Using map-based cloning, *GmCCD4* was isolated and functionally characterized. It can degrade carotenoid into β-ionone, and it is a negative regulator of carotenoid accumulation [32]. Recently, a study in gardenia reveals that over-expression of *GjCCD4a* can significantly reduce the content of colored carotene and xanthophylls [33].

The mechanisms by which transcription factors (TFs) regulate the carotenoid biosynthesis structural genes have also been illustrated over the last several years. *GLK1* and *GLK2*, GARP subfamily MYB TFs, can alter the numbers and activity levels of plastids to positively regulate the biosynthesis of various carotenoids, such as octahydro-lycopene, lutein, and lycopene [34]. Conversely, MYB TFs can reduce carotenoid accumulations in papaya pulp [35] and kiwifruit [36–38]. MADS is a common TF that affects the carotenoid synthesis pathway. *MADS-RIN* [39], *SlMBP15* [40], *SlCMB1* [41], *CsMADS5* [42], *CsMADS6* [43], and *CsMADS3* [44] are positive regulators, whereas *SlMBP8* [45] and *SlFYFL* [46] are negative regulators. Among NAC TFs, *SlNAC1* and *SlNAC9* negatively regulate carotenoid synthesis [47, 48], whereas *SlNAC4* positively regulates carotenoid synthesis [49]. In papaya, *CpNAC1* and *CpNAC2* may act as positive regulators of carotenoid biosynthesis, possibly through the transcriptional activation of carotenoid biosynthesis-related genes [50, 51]. In sweet potato, *IbNAC29* was very recently found to be a positive regulator of carotenoid accumulation [52]. Another important TF, ERF associated with ethylene, also plays roles in the carotenoid biosynthesis pathway. In tomato, *SlAP2a* [53] and *SlERF6* [54] weaken carotenoid biosynthesis by regulating the ethylene synthesis pathways during fruit ripening. *MdAP2*–*34* promotes carotenoid accumulation in *MdAP2–34-OVX* transgenic apple calli and fruit by participating in the carotenoid biosynthesis pathway, and it regulates phytoene and β-carotene, but not lutein, accumulations. *MdPSY2*–1 is a major gene in the carotenoid biosynthesis pathway in apple fruit, and it is directly bound and transcriptionally activated by *MdAP2–34* in apple calli, resulting in increased phytoene and total carotenoid contents [55]. Zhu *et al*. [56] demonstrated that carotenoid accumulation was enhanced by increasing the expression of *LCYb2* via ERF TFs, and *CsERF061* directly binds to the promoter of *LCYb2* and activates its expression in citrus and tomato. Recently, Wang *et al*. [57] reported that *CitERF23* showed significant positively correlation with *CCD4*, indicating that ERF family played a role in regulating carotenoid metabolism in pummelo. MaERF124 acts as a transcriptional repressor and negatively modulates carotenoid accumulation during banana’s fruit ripening [58].

Ethylene has been reported to be involved in the regulation of carotenoid accumulation [56, 58, 59], and the corresponding usually activated ERF TF or TF complex regulates carotenoid production in plants. We have reported that *CCD4.1* might have a potential relationship with *ERF5* [60], but whether ERFs and *CCD4.1* are involved in regulating carotenoid accumulation remains unclear in wolfberry. In the present study, we isolated *LbCCD4.1* through CCD family expression during fruit development and correlations with carotenoids, followed by function characterization using a transient over-expression assay. This paper verified that *LbERF5.1* can bound to the promoter of *LbCCD4.1*, enhancing its expression, which might accelerate carotenoid degradation. These results provided new insights into the regulation of carotenoid accumulation in wolfberry’s fruit.

**Figure 1:**
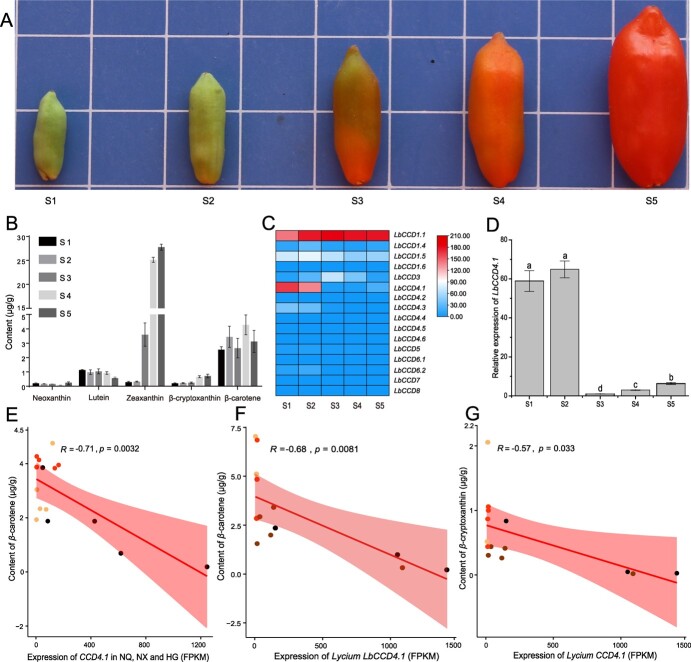
*LbCCD4.1* was associated with the carotenoid content. **A** Appearance of *Lycium barbarum*’s fruit at five key developmental stages (S1–S5). **B** Dynamics of carotenoid metabolites in NQ fruit during five key developmental stages, and the histograms of every metabolite from left to right indicated S1, S2, S3, S4, and S5, respectively. **C** Heatmap of quantitative expression dynamics of *LbCCD* family genes in NQ fruit at the five key developmental stages based on transcriptome sequencing. **D** Relative expression of *LbCCD4.1* in NQ fruit during the five key developmental stages. **E** Correlations between the β-carotene content and *CCD4.1* expression among NQ (red dot), NX (yellow dot), and HG (black dot) fruits during stage S1 to S5. **F** and **G** Correlations between the β-cryptoxanthin content, β-carotene content, and *CCD4.1* expression among 14 *Lycium* accessions. The 14 *Lycium* included four NQ (red dot), two NX (yellow dot), three HG (black dot), and five other types (brown dot). The expression and metabolite content measurements included three biological replicates, and values are presented as averages ± standard deviations. Multiple comparisons were performed using one-way ANOVA and Tukey’s multiple range test (*P* < 0.05).

**Figure 2:**
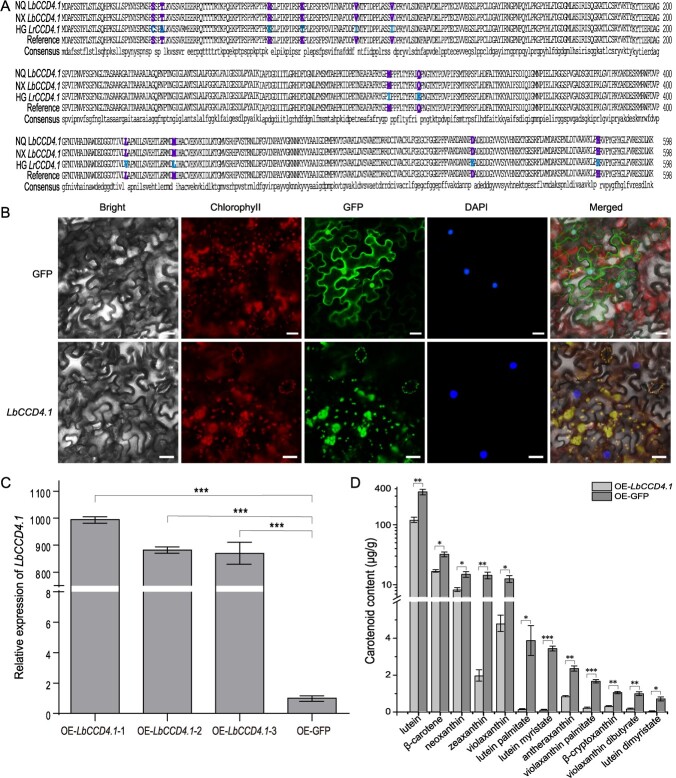
Sequence analysis and functional verification of *LbCCD4.1*. **A** Multiple sequence alignment of the *CCD4.1* genes in NQ, NX and HG. **B** Subcellular localization of *LbCCD4.1* in tobacco leaves. Scale bars = 50 μm. **C** The qRT-PCR detection of *LbCCD4.1* expression in the leaves of NQ OE-*LbCCD4.1* plants using GFP as the control. **D** Detection of carotenoid metabolites in the leaves of NQ *LbCCD4.1* over-expression plants. Three biological replicates were used, and values are presented as averages ± standard deviations. Student’s *t*-test was used to determine significance: ^*^P < 0.05; ^**^P < 0.01; ^***^P < 0.001.

## Results

### *LbCCD* family identification and expression profile characterization

The fruit color of ‘Ningqi No.1’ (NQ) gradually changes from green to yellow-green and then to red during the ripening process (Fig. 1A). Fruit samples at five key developmental stages (from S1 to S5) were used for the quantitative analysis of five carotene metabolites. The zeaxanthin kept a relatively low content during stage S1 to S2 but was the highest at stage S5, whereas the neoxanthin remained at a low content during the whole developmental period. β-cryptoxanthin gradually accumulated to a stabilized level from the S1 to S5; and β-carotene gradually accumulated through the S4 stage and then decreased during the S5 stage, and lutein kept a relatively stable content from S1 to S4 but a significant decrease at S5, indicating that β-carotene and lutein might be degraded to a certain extent during late development (Fig. 1B; Table S1, see online supplementary material). On the basis of the *Lycium* genome sequence, 18 *L. barbarum* carotenoid cleavage dioxygenase (*LbCCD*) genes were identified. Among the *LbCCD* family genes, *LbCCD4.6* was the longest at 31358 bp and *LbCCD4.4* was the shortest sequence at 818 bp. *LbCCD* family protein sequences ranged from 171 to 644 aa, and the molecular weights were between 18742.55 and 72607.61 Da (Table S2, see online supplementary material). A phylogenetic analysis (Fig. S1, see online supplementary material) showed that these genes could be divided into three groups: Class I included *LbCCD7* and *LbCCD8*, which were mainly involved in regulating the growth and development of lateral branches; Class II included *LbCCD1* and *LbCCD4*, which were mainly involved in the formation of flavor and volatiles; and Class III included *LbCCD*3, *LbCCD*5, and *LbCCD6*, which were mainly involved in the biosynthesis of abscisic acid (ABA) [61, 62]. In addition, the *LbCCD* family genes were highly similar to those in *Arabidopsis*, tobacco, and tomato, indicating that *LbCCD*s were conserved during species differentiation (Fig. S1, see online supplementary material). Using transcriptome data from the fruits of NQ at stage S1, S2, S3, S4, and S5, only two of the 18 genes were not detected (Fig. 1C). The expression of *LbCCD4.1* was higher in the S1 and S2 stages, decreased rapidly after the S3 stage, and then increased slightly in the S5 stage (Fig. 1D). A further correlation analysis using the expression of *CCD4.1* and the β-carotene content in the fruits of NQ, NX, and HG from stage S1 to S5 revealed that *CCD4.1* expression was extremely negatively correlated with the β-carotene level (R = −0.71, *P* = 0.0032) (Fig. 1E). Finally, we used a total of 14 *Lycium* accessions (fruits at stage S5) to explore the correlation between *CCD4.1* and carotenoid content. Similarly, extremely negative correlations were observed between *Lycium CCD4.1* expression and both β-carotene and β-cryptoxanthin levels (Fig. 1F and G). These results indicated that *LbCCD4.1* might be an important gene involved in carotenoid metabolism.

### Functional characterization of *LbCCD4.1*

We cloned the coding sequences (CDSs) of CCD4.1 in NQ, NX, and HG, of which the colors of full ripe fruit were red, orange-yellow, and black, respectively (Fig. 1A; Fig. S2, see online supplementary material). The ORF of the *CCD4*.1 gene was 1800 bp, encoding 599 aa, in each of the three accessions. A multiple sequence alignment showed that the CDSs of *LbCCD4.1* in NQ and NX were completely consistent, but that of the *LrCCD4.1* in HG had 31 SNP variants, resulting in sequence differences of 12 aa (Fig. 2A; Fig. S3, see online supplementary material), which suggested that *CCD4.1* was conserved in the same species, but had differentiated among species. We constructed a GFP fusion marker expression vector containing *LbCCD4.1* (Fig. S4, see online supplementary material), and a transient expression analysis was performed to detect the subcellular localization of *LbCCD4.1* in tobacco leaves. *LbCCD4.1* was localized on the chloroplast (Fig. 2B). Furthermore, an *LbCCD4.1* transient over-expression (OE) vector (Fig. S5, see online supplementary material) was constructed and transformed into NQ’s leaves. The expression of OE-*LbCCD4.1* plants was significantly increased (Fig. 2C). Carotenes and xanthophylls, such as β-carotene and β-cryptoxanthin, respectively, in the OE-*LbCCD4.1* plants were significant or extremely significant down-regulated (Fig. 2D). Thus, *LbCCD4.1* negatively mediated the accumulation of carotenoid metabolites.

### Identification and genetic variations of *LbERF5.1*

**Figure 3:**
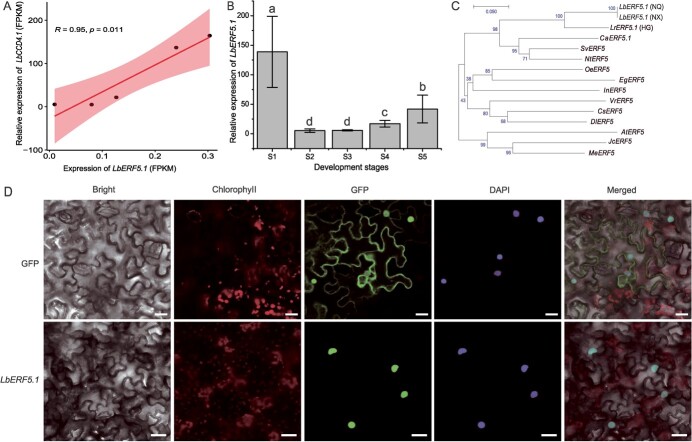
Identification of *LbERF5.1*. **A** Correlation between *LbCCD4.1* and *LbERF5.1* expression levels in NQ fruit at five key developmental stages. **B** Expression profile of *LbERF5.1* in NQ fruit at five key developmental stages. Multiple comparative analyses were performed using one-way ANOVA and Tukey’s multiple range test (*P* < 0.05). **C** Evolutionary tree analysis of ERF5.1 in 14 diverse species. The evolutionary tree was constructed using the Neighbor Joining method of MEGA7 with bootstrap = 1000. **D** Subcellular localization of *LbERF5.1* in tobacco leaves. Scale bars = 50 μm.

The promoter of *LbCCD4.1* contains three predicted binding sites (Fig. S6, see online supplementary material), which may be the TF recognition sites of ethylene responsive factor (ERF). We identified the ERF TF expressed in the fruit of NQ at S5 stage, *LbERF5.1*, and a screen determined that its expression was significantly positively correlated with *LbCCD4.1* expression (R = 0.95, *P* = 0.011) (Fig. 3A). The *LbERF5.1* expression was higher at early stages of fruit development, then decreased rapidly, and finally increased slowly at later stages. The expression pattern was consistent with that of *LbCCD4.1* (Fig. 3B). The ORF of *LbERF5.1* in NQ was 735 bp, encoding 244 aa, including 35 basic aa (Asp+Glu) and 36 acidic aa (Arg + Lys). In addition, we cloned the *LrERF5.1* in HG, and the length was 873 bp, encoding 290 aa, including 41 basic aa (Asp+Glu) and 39 acidic aa (Arg + Lys). An evolutionary tree analysis (Fig. 3C) of 14 diverse species revealed that the ERF5s of Solanaceae species clustered into a single group. Three *Lycium* ERF5.1 grouped into one branch, with *CaERF5* being the closest to *LbERF5.1*. Furthermore, we constructed a GFP fusion vector (Fig. S7, see online supplementary material) and transiently over-expressed it in tobacco leaves. The GFP and DAPI nuclear staining signals were coincident, indicating that *LbERF5.1* was localized in the nucleus (Fig. 3D).

**Figure 4:**
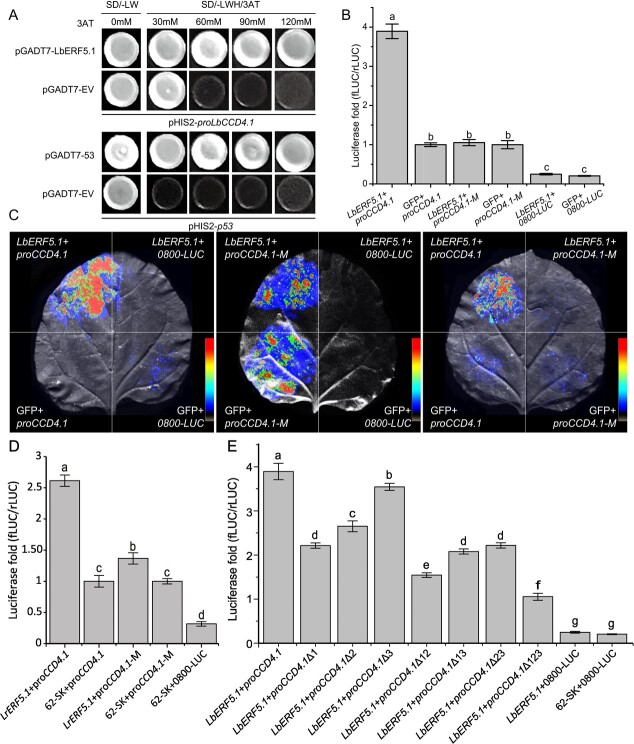
Physical interaction between *CCD4.1* and *ERF5.1*. **A** A yeast one-hybrid experiment showed that *LbERF5.1* bound to the promoter region of *LbCCD4.1*. The pGADT7-p53 and pHIS2-p53 were used as positive quality controls, and pGADT7 and pHIS2-p53 were used as negative quality controls. The pGADT7 and pHIS2-pro*LbCCD4.1* served as negative controls. **B** and **C** A dual-luciferase assay showed that *LbERF5.1* enhanced the promoter activity of *LbCCD4.1*. **D** A dual-luciferase assay showed that *LrERF5.1* enhanced the promoter activity of *LrCCD4.1*. **E** A dual-luciferase assay showed *LbERF5.1*’s binding activity with the three predicted binding sites of *LbCCD4.1*’s promoter. Luciferase fold-changes in tobacco under different effector and reporter gene combinations were calculated using the ratio of firefly luciferase to renal luciferase (fLUC/rLUC). All the data were calculated as the mean values of three replicates. Error bars represent standard deviations. The data were analysed using one-way ANOVA and Tukey’s multiple range test (*P* < 0.01).

### Interaction between *ERF5.1* and *CCD4.1*

The yeast one-hybrid (Y1H) experiment showed that all Y187 strains co-transfected with pGADT7 and pHIS2 plasmids produced clones on the double amino acid-deficient medium. Yeast co-transformed with plasmids containing *LbERF5.1* and *LbCCD4.1* promoters grew normally in SD/-His/−Leu/−Trp plates with a high concentration of 3-amino-1,2,4-triazole, whereas no clones grew from the negative control group, which indicated that *LbERF5.1* bound to the promoter sequence of *LbCCD4.1 in vivo* (Fig. 4A). To further explore the specific transcriptional regulation of *LbERF5.1* on *LbCCD4.1*, we recombined *LbERF5.1* into the pGreenII-62-SK vector and the *LbCCD4.1* promoter into the dual-luciferase vector pGreenII0800-LUC. Three binding site-deleted mutations in *LbCCD4.1*’s promoter formed proCCD4.1-M, which served as a control (Fig. S8, see online supplementary material). When the system did not contain *LbCCD4.1*’s promoter, there was almost no expression of the Luc reporter gene; however, when the system contained *LbCCD4.1*’s normal promoter, the Luc reporter gene was expressed (Fig. 4B and C), indicating that the *LbCCD4.1* promoter sequence had activity. The promoter activities of mutant proCCD4.1-M and wild-type pro*LbCCD4.1* were not obviously different, indicating that mutating the promoter did not change its activity. In addition, when *LbERF5.1* was over-expressed in the system, *LbERF5.1* recognized the promoter and the downstream reporter gene Luc was expressed. However, when pro*LbCCD4.1*-M was in the system, *LbERF5.1* did not promote the expression of the downstream reporter gene Luc, indicating that the deletion of pro*LbCCD4.1* promoter site resulted in *LbERF5.1*’s inability to bind, which led to a lack of downstream reporter gene Luc expression, indicating that *LbERF5.1* could bind to the promoter of *LbCCD4.1* and activate its expression (Fig. 4B and C). To test the bind activity of *LrCCD4.1* and *LrERF5.1* (*Lycium ruthenicum*), we conducted the dual-luciferase assay using the same system in HG. Similarly, *LrERF5.1* could bind to the promoter of *LrCCD4.1* (Fig. 4D). To further investigate whether the transcription factor *LbERF5.1* could specifically bind to the three binding sites in the *LbCCD4.1*’s promoter region, we constructed single, double, and triple mutation vectors for dual-luciferase experiments (Fig. S9, see online supplementary material). The results showed that the luciferase fold (fLUC/rLUC) of wild-type had the highest detection value, while single mutations △1, △2, and △3 performed significantly reduced activity compared with the wild-type but were still significantly higher than that in the control, indicating that all the three sites had binding activity with *LbERF5.1* but with different promoter activity abilities (△1 > △2 > △3). This was consistent with the results of double mutations, where △12 had significantly lower promoter activity compared with △23 and △13. The binding activity of the triple mutation △123 was significantly lower than that in the wild-type and either single or double mutations (Fig. 4E).

### Functional verification of *ERF5.1* in carotenoid accumulation

**Figure 5:**
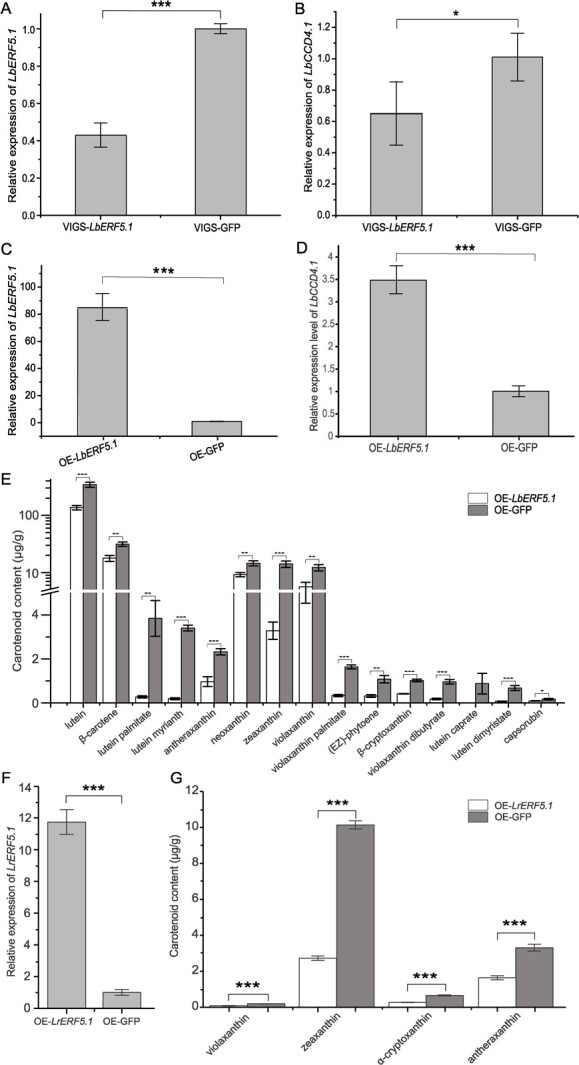
*ERF5.1* promoted the expression of *CCD5.1* and participated in the regulation of carotenoid accumulation. **A** Expression of *LbERF5.1* and GFP under VIGS of *LbERF5.1* conditions as assessed by qRT-PCR. **B** Expression of *LbCCD4.1* and GFP under VIGS of *LbERF5.1* conditions as assessed by qRT-PCR. **C** The levels of *LbERF5.1* in *LbERF5.1* over-expression plants as assessed by qRT-PCR. **D** The levels of *LbCCD4.1* in *LbERF5.1* over-expression plants as assessed by qRT-PCR. **E** Detection of carotenoid metabolites in the leaves of NQ *LbERF5.1* over-expression plants. **F** The expression levels of *LrERF5.1* in *LrERF5.1* over-expression plants (NQ’s leaves) as assessed by qRT-PCR. **G** Detection of carotenoid metabolites in the leaves of OE-*LrERF5.1* over-expression plants (NQ’s leaves). All assays contained three biological replicates. Student’s *t*-test was used for significance comparisons: ^*^P < 0.05; ^**^P < 0.01; ^***^P < 0.001.

To verify the function of *LbERF5.1* in the regulation of carotenoid biosynthesis, we firstly tested the expression patterns in the fruits of NQ, NX, and HG at S5 stage. The results showed that the *ERF5.1* expression in NQ was the lowest, followed by in NX, and the highest was in HG with extremely significant differences (Fig. S10, see online supplementary material), the trend of which was opposite to the carotenoids content of a report [63], indicating its negative regulation role. We further constructed OE (Fig. S11, see online supplementary material) and virus-induced gene silencing (VIGS) vectors, respectively, (Fig. S12, see online supplementary material) and carried out the transient transformation of NQ’s leaves. The expression of *LbERF5.1* in VIGS plants decreased significantly (Fig. 5A), as did the expression of *LbCCD4.1* (Fig. 5B). In OE plants, the expression of *LbERF5.1* was significantly higher than that of GFP (*P* < 0.001) (Fig. 5C). In addition, the expression of *LbCCD4.1* was also significantly increased (Fig. 5D). The carotenoid contents, especially lutein, violaxanthin and their derivatives, in OE plants were significantly or extremely significantly down-regulated (Fig. 5E). Moreover, the β-carotene content decreased significantly in OE plants. Thus, *LbERF5.1* appeared to positively regulate *LbCCD4.1* expression and then negatively regulate carotenoid metabolite accumulation. As the carotenoid content was low and the expression of *ERF5.1* was high in HG, we decided to transfer *LrERF5.1* to NQ’s leaves because of the small leaves hampered transient transformation assay in HG (Fig. 5F). Analogously, some carotenoid metabolites (violaxanthin, zeaxanthin, α-cryptoxanthin, and antheraxanthin) were down-regulated in the OE-*LrERF5.1* plants (Fig. 5G).

### Over-expressing *LbERF5.1* and *LbCCD4.1* affects the global transcriptome and contributes to the carotenoid biosynthetic pathway in transgenic *Lycium.*

We performed an RNA-seq analysis of over-expressing *LbCCD4.1* and *LbERF5.1* leaves in NQ. Compared with the control, there were 9751 differentially expressed genes (DEGs) in OE-*LbCCD4.1* plants, of which 5273 were up-regulated and 4478 were down-regulated. In OE*-LbERF5.1* plants, there were 7651 DEGs, of which 4417 were up-regulated and 3234 were down-regulated. A total of 5967 DEGs were shared between *LbCCD4.1* and *LbERF5.1* transformed plants (Tables S3and S4; Fig. S13, see online supplementary material). Among the DEGs in *LbCCD4.1* and *LbERF5.1*, 511 and 409 annotated genes were TFs, respectively. The most differently expressed regulatory genes were in the AP_2_/ERF, WRKY, MYB, NAC, C_2_H_2_ and bHLH families (Tables S3and S4). A GO analysis indicated that the DEGs in the comparisons of *LbCCD4.1* vs. control and *LbERF5.1* vs. control were similarly enriched in the top three terms, photosynthesis, calmodulin binding, and apoplast (Fig. S14, see online supplementary material). A KEGG analysis indicated that the DEGs in the comparisons *LbCCD4.1* vs. control and *LbERF5.1* vs. control were all principally associated with the metabolic pathway and biosynthensis of secondary metabolites (Fig. S14, see online supplementary material).

In OE-*LbCCD4.1* and OE-*LbERF5.1* plants, the upstream genes of the carotene biosynthesis process were differentially expressed to different degrees. Among the *GGPS* genes, *evm.TU.chr02.3627* and *evm.TU.chr11.272* were down-regulated by approximately 5.0 times in OE-*LbCCD4.1* plants, and by approximately 2.5 times in OE-*LbERF5.1* plants. Notably, the expression of evm.TU.chr10.291 increased sharply by 42 times in OE-*LbCCD4.1* plants and 72.9 times in OE-*LbERF5.1* plants. *PSY* genes (*evm.TU.chr01.2052*, *evm.TU.chr11.3124*, and *evm.TU.chr12.1296*) were significantly down-regulated in OE-*LbCCD4.1* plants, whereas one *PSY* gene, *evm.TU.chr11.3124*, was up-regulated in OE-*LbERF5.1*, which indicated that the over-expression of *LbCCD4.1* and *LbERF5.1* could change the expression of rate-limiting enzymes, thereby affecting the accumulation of upstream substances in carotenoid synthesis. In the downstream pathway of carotenoid biosynthesis, *LCYE* (*evm.TU.chr04.3738*), *CYP97A* (*evm.TU.chr10.1526*), *CYP97C* (*evm.TU.chr08.2566*), and *ZEP* (*evm.TU.chr03.2986*) were significantly down-regulated (2–3 times) in OE-*LbCCD4.1* and OE-*LbERF5.1* plants, whereas *ZEP* (*evm.TU.chr12.3134*) was only down-regulated 2.5 times in OE-*LbERF5.1* plants. The expression of *ZEP* (*evm.TU.chr07.2471*) in OE-*LbCCD4.1* and OE-*LbERF5.1* plants was significantly enhanced relative to the wild type, by up to 44.1 times and 29.4 times, respectively. However, the expression of *PDS*, *ZISO*, *ZDS*, *CriISO*, *LCYB*, and *BCH* did not change significantly. Thus, *LbCCD4.1* and *LbERF5.1* appeared to have similar effects on the overall expression level and on the carotene gene-specific expression in transgenic *Lycium* leaves.

## Discussion

Carotenoids are a series of important secondary metabolites that function mainly in the growth and development fruit of plants. *CCD*s are structural genes required for carotenoid degradation [23, 24] that may be involved in regulating carotenoid accumulation in *Lycium*. In the present study, we identified the *LbCCD4.1* gene *in L. barbarum*, and we also identified *LbERF5.1*, an ERF type TF that interacts with the promoter region of *LbCCD4.1*. We functionally characterized *LbCCD4.1* and *LbERF5.1* as being involved in significantly regulating the accumulation of different carotenoid metabolites in *Lycium*.

### *LbCCD4.1* decreased carotenoid metabolite accumulation in *Lycium.*

**Figure 6:**
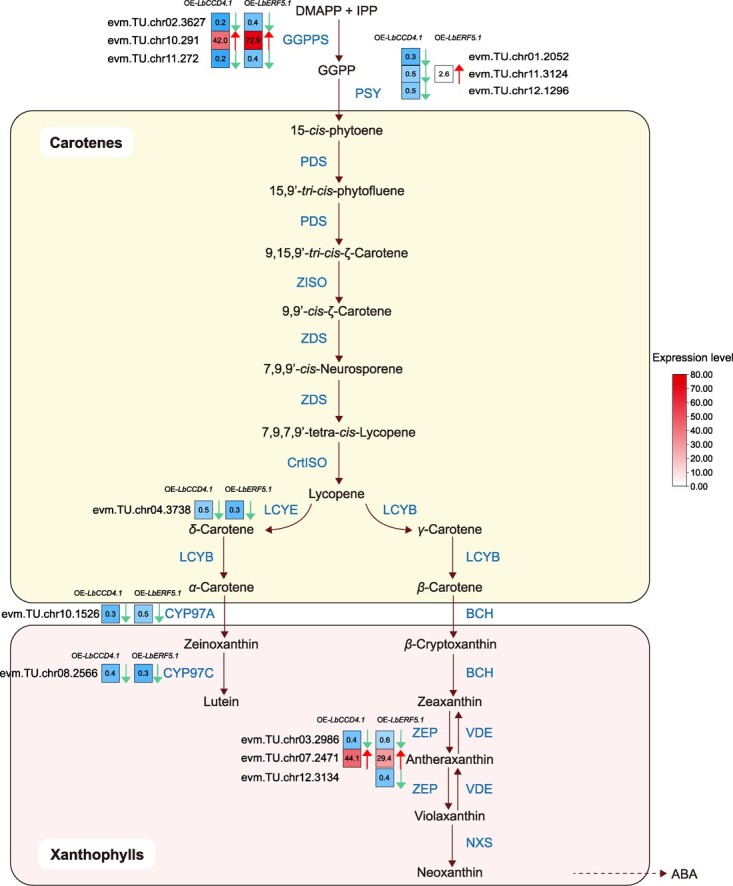
Differential expression of carotenoid pathway-involved genes induced by over-expressing *LbCCD5.1* and *LbERF5.1*. Solid and dashed arrows indicate direct and indirect reaction flows in the pathway, respectively. The enzymes encoded by the related DEGs in the carotenoid pathway are located next to the arrows. The left and right adjacent square heat maps represent the corresponding DEGs in OE-*LbCCD4.1* and OE-*LbERF5.1* plants, respectively, with fold-change (OE-plants/CK) values. The green-down and red-up arrows indicate significant decreases and increases, respectively. ABA, abscisic acid.

An analysis of *CCD4*.1 sequences from HG (*Lycium ruthenicum* Murr.), NQ (*L. barbarum* Linn.), and NX (*L. barbarum* Linn.) wolfberry showed that the sequences of the latter two were the same, whereas that of the former was slightly different. The gene expression patterns of *CCD4*.1 in red fruit and black fruit wolfberry differed, with the expression in the latter being higher than in the former [6], and the latter also had a low carotenoid content [5], suggesting that *CCD4*.1 was conserved in the same species of *Lycium* but had undergone potential functional differentiation among species. Furthermore, we identified 11 SNPs in the promoter region (~2 kb upstream) of *CCD4.1* in NQ, NX, and HG (Fig. S15, see online supplementary material). However, no SNPs were located in the three predicted binding sites of *ERF5.1*, indicating that these SNP may not affect the binding ability. In *Lycium chinense*, the amino acid sequence of *LcCCD4* shares homology with that of CCD4 proteins from other *Solanaceae* plants [64], further confirming its conservation. The *LbCCD4.1* expression was associated with wolfberry fruit ripening and was significantly negatively correlated with the carotenoid metabolite accumulations in fruit (Fig. 1). What should be noted was that although the expression level of *LbCCD4.1* was the highest in the stages of S1 and S2 (Fig. 1D) and the carotenoid content was low in these two stages, a lower content of carotenoids might be caused by lower biosynthesis but not by higher degradation by higher expression of *CCD4.1* during the early development stages of goji fruit. Furthermore, over-expressing *LbCCD4.1* in NQ leaves resulted in a reduction in carotenoid profiles (Fig. 2) and the corresponding expression of geranylgeranyl diphosphate synthase, PSY, LCYE, CYP97A, and CYP97C (Fig. 6), indicating that *LbCCD4.1* affected carotenoid accumulation through changing global carotenoid biosynthesis. *DcCCD4* affected the accumulation of carotenoids through clearance of α-carotene and β-carotene in carrot taproot [29]. Here, the over-expressing *LbCCD4.1* did not demonstrate a change in the α-carotene content, whereas the β-carotene content decreased, suggesting that *LbCCD4.1* only acted on the latter. Nevertheless, further direct evidence is needed to determine which *LbCCD4.1* enzyme can cleave β-carotene.

### *LbERF5.1* is a positive regulator of *LbCCD4.1* involved in carotenoid metabolism.

We determined that *LbERF5.1* binds to the promoter of *LbCCD4.1* (Fig. 4) using Y1H and dual-luciferase assays, and a typical motif [65, 66] promoted the expression of *LbCCD4.1* in OE-*LbERF5.1* transgenic lines (Fig. 5D). In the OE-*LbERF5.1* lines, *LbCCD4.1* was up-regulated, in accordance with its negative role in carotenoid, especially lutein and violaxanthin accumulation (Fig. 5E). *LbCCD4.1* expression was expectantly inhibited in the *LbERF5.1*-silenced lines, which was in line with the expression pattern of an AP_2_/ERF TF, *MdAP2–34*, in relation to *MdCCD4* in the fruit skin of apples [55]. However, *MdAP2*–*34* promotes phytoene and β-carotene, but not lutein, accumulations [55], suggesting that *LbERF5.1* and *MdAP2–34* have different roles in the carotenoid biosynthesis pathway. Moreover, the binding activity between *CCD4.1* and *ERF5.1* in HG could also be detected (Fig. 4D), indicating that similar regulatory mechanism in carotenoid accumulation existed between *L. barbarum* and *L. ruthenicum.* In addition, the reduced expression of ERFs can regulate carotenoid biosynthesis by enhancing both carotenoid and ethylene levels during fruit ripening [54, 67] and by directly binding to the structural genes required for carotenoid biosynthesis, such as *PSY* [55] and *LCYb2* [56] to regulate carotenoid accumulation. Therefore, to further understand the mechanisms of carotenogenesis in wolfberry, we will identify the direct targets of *LbERF5.1* using other assays, such as chromatin immunoprecipitation sequencing analysis in the future.

### *LbCCD4.1* and *LbERF5.1* can affect multiple carotenoid synthesis pathway genes.

Silencing carotenoid biosynthesis structural genes, like *PDS*, *ZDS*, *βOH*, *ZEP*, and *PSY*, causes a decrease in the total carotenoid contents [68–70]. In the OE-*LbCCD4.1* and OE-*LbERF5.1* plants, we also detected that some carotenoid biosynthesis structural genes were down-regulated (Fig. 6), which may partially explain the decline in carotenoid accumulation. However, the expression levels of two genes in OE lines, *evm.TU.chr10.291* and *evm.TU.chr07.2471*, encoding geranylgeranyl diphosphate synthase and *ZEP*, respectively, were dramatically up-regulated by *LbCCD4.1* and *LbERF5.1*. Thus, a complex inducement mechanism of carotenoid biosynthesis genes may affect carotenoid accumulation, which was similar to a previous report in sweet orange [43]. Notably, the RNA-seq analysis results suggested the involvement of the AP_2_/ERF, WRKY, MYB, NAC, C_2_H_2_, and bHLH families in the regulation of *Lycium* carotenoid compound accumulation (Tables S3 andS4, see online supplementary material), indicating possible critical links among different TFs in the regulation of carotenoid biosynthesis, and even fruit ripening, in *Lycium*, as reported by a previous report on the light-specific regulatory mechanism of carotenoid biosynthesis in rice leaves [71].

## Materials and methods

### Plant materials and fruit sampling

The experimental accessions were from the National Wolfberry Engineering Research Center of China (38°380′N, 106°9′E; altitude 1100 m). The fruits of a wide genetic range of 14 eight-year old *Lycium* accessions involving over five species (Table S5, see online supplementary material), including NQ (*L. barbarum* L.), NX (*L. barbarum* L.), and HG (*L. ruthenicum* Murr.) at five key developmental stages, 9–12 (S1), 14–19 (S2), 20–26 (S3), 30–37 (S4), and 38–45 (S5) days post-flowering, were sampled with 20 biological repetitions and quickly frozen in liquid nitrogen. They were then stored at −80°C for transcriptome, metabolome, and quantitative real-time PCR (qRT-PCR) experiments. Hydroponic seedlings of NQ were used for transient over-expression (OE) and VIGS experiments.

### Carotenoid contents determination

The carotenoids extractions and content determinations were performed as described previously [72].

### C‌CD family identification and sequence analysis

First, CCD family genes were selected as queries from the Arabidopsis genome sequences in line with the report of Tan *et al.* [62]. Second, BLASTP was used to identify the best match in the genome of *Lycium* (NQ) with threshold value E < 1e^−10^. Thereafter, hmmer [73] was used to search for the RPE65 domain of CCD genes among the hit genes using E < 1e^−5^ as threshold value. Finally, the above hit genes were mapped in SMART [74], CDD [75], Gene3D [76], and PRINTS [77] databases using InterProScan [78] with default parameters. The candidates from all the four databases were used to generate CCD family gene set in wolfberry. ExPASy [79] and WoLF PSORT (https://wolfpsort.hgc.jp/) were used to predict the physicochemical properties and subcellular localizations of the CCD genes. MEGA7 [80] was used for Neighbor Joining phylogenetic tree construction (bootstrap = 1000).

### Identification of carotenoid pathway genes in *Lycium*

To identify homologous genes of the carotenoid pathway in *Lycium*, we downloaded the protein sequences of reported carotenoid pathway structural genes and TFs from the NCBI and TAIR [81] databases. These protein sequences were used as query to search against the protein annotations of *Lycium*, and the putative proteins were obtained by BLASTP searching (E < 1e^−10^). In addition, the putative proteins were submitted to the Pfam database to identify conserved domains having E = 1.0, and proteins without corresponding conserved domains were excluded from further analysis.

### Gene and promoter cloning in *Lycium*

Using *Lycium* fruit as test material, total RNA was isolated using a RNA extraction kit from Takara (Takara, Dalian, China). Single-strand cDNA of *CCD4.1* and *ERF5.1* were prepared using a Reverse Aid First-strand cDNA Synthesis Kit (Thermo Fisher Scientific, Waltham, MA, USA). Using the *Lycium* genome and transcriptome data, primers (Table S6, see online supplementary material) were designed [82] to amplify the CDSs of *CCD4.1* and *ERF5.1* with the following system component: 25 μL 2× PCR Buffer, 10 μL dNTP (2 mM), 2 μL upstream primer (10 μM), 2 μL downstream primer (10 μM), 5 μL single-stranded cDNA, 1 μL KOD FX Neo (Toyobo Life Science, Osaka, Japan) and 10 μL Millipore H_2_O. The PCR reaction procedure was set as: pre-denaturation at 98°C for 3 min, and followed by 30 cycles of denaturation for 10 s, 58°C annealing for 30 s, 68°C extension for 2 min; and final extension at 68°C for 5 min. The amplified products were purified, and independently cloned onto the pMD18-T vector (TaKaRa, Tokyo, Japan) for Sanger sequencing. The 2000-bp upstream sequence of the *CCD4.1* was treated as a possible promoter sequence. A *cis*-element was predicted using PlantCARE [83]. DNA from NQ, NX and HG’s fruits were extracted using the hexadecyl trimethyl ammonium bromide method. The primers (Table S6, see online supplementary material) were designed to amplify the promoter sequence of the *CCD4.1* with the same primer3 procedure. The PCR cloning reaction system was basically the same as *CCD4.1* and *ERF5.1* cloning.

### Subcellular localization assay

*CCD4.1* and *ERF5.1* were cloned into the tobacco OE vector pCambia1300–35 s-GFP independently, and transferred into *Agrobacterium* GV3101. The infective bacterial solution was obtained using infection buffer (5 g/L D-glucose, 50 mM MES, 2 mM Na_3_PO_4_·12 H_2_O and 0.1 mM acetobutanone) at an OD_600_ = 1.0 at 20–25°C for 1–2 h. At 72 h after injection into the tobacco leaves with the bacterial solution, fluorescence signals were visualized using laser copolymerization fluorescence microscopy. GFP and DAPI excitation were determined at 488 nm and 405 nm, respectively.

### Over-expression and VIGS transformation

To obtain OE lines of *Lycium*, the full-length cDNAs of *LbCCD4.1*, *LbERF5.1*, and *LrERF5.1* were amplified. Then, the cDNAs were independently cloned into the pCambia 1300–35 s vector to obtain the p35s: *LbCCD4.1*, p35s: *LbERF5.1* vectors and p35s: *LrERF5.1* vectors, respectively. The OE vector was transformed into *Agrobacterium* GV3101 in infection buffer solution (5 g/L D-glucose, 50 mM MES, 2 mM Na_3_PO_4_·12 H_2_O and 0.1 mM acetobutanone) at an adjusted OD_600_ = 1.0 at 20–25°C for 1–2 h. The infective bacterial solution was injected into NQ leaves to obtain OE-*LbCCD5.1*, OE-*LbERF5.1*, and OE-*LrERF5.1* plants. The pCambia1300–35 s-GFP was used as the negative control. Samples were taken at 72 h after injection for qRT-PCR, metabolome, and RNA-seq analyses, with each sample having three biological repetitions.

For VIGS assay, 366 bp of the *LbERF5.1* (1–366) region was amplified and cloned into the pTRV2 vector. The primers are shown in Table S6 (see online supplementary material). The vector pTRV2-*LbERF5.1* was transformed into *Agrobacterium* GV3101 as described above. The NQ leaves were transfected by *Agrobacterium* infiltration. The bacterial solution containing the pTRV2 empty carrier was used as a negative control, and pTRV2-GFP was used as a positive control. Fresh leaves of three lines were collected for qRT-PCR testing after transformation 7–10 days.

### Yeast one-hybrid (Y1H) assay

The *LbCCD4.1* promoter, containing the GCC-box, was cloned and fused to the HIS3 mini-promoter in the pHIS2 vector to obtain the reporter construct. The full-length *LbERF5.1* was fused to the GAL4 activation domain in pGADT7, which was co-transformed with the reporter construct into the yeast strain (Y187) in 600 μL of PEG/LiAc solution. The pGADT7–53 and pHIS2-P53 were co-transformed as positive controls, whereas pGADT7 + pHIS2-P53 and pGADT7 + pHIS2-pro*LbCCD4.1* were used as negative controls. The yeast cells were transferred into SD/−Leu/−Trp medium for positive clone selection. Then, the DNA-protein interaction was surveyed by the appearance of yeast’s growing status on SD/−Trp/−Leu/-His medium supplemented with 30 mM, 60 mM, 90 mM, and 120 mM concentrations of 3-amino-1,2,4-triazole independently.

### Dual-luciferase assay

The possible recognition motifs in the *LbCCD4.1* promoter (proCCD4.1) and *LbERF5.1* TF were predicted using the Jaspar database (https://jaspar.genereg.net/). A total of three possible binding sites, located at −44, −116, and – 277 bp upstream of the TSS (Fig. S6, see online supplementary material), were identified. A total of three combinations, *LbERF5.1* & *LbCCD4.1*’s promoter, *LrERF5.1* & *LrCCD4.1*’s promoter, *LbERF5.*1 & three binding site of *LbCCD4.1*’s promoter, were designed to illustrate their binding activities. For combination *LbERF5.1* & *LbCCD4.1*’s promoter, a mutant promoter of proCCD4.1 (proCCD4.1-M) and *LbCCD4.1* were cloned into the dual luciferase vector pGreenII 0800-LUC using homologous recombination methods. The mutant promoter proCCD4.1-M was used as a negative control, and *LbERF5.1* was homologously recombined onto the pGreenII-62-SK vector. The similar experimental operation was conducted to combination *LrERF5.1* & *LrCCD4.1*’s promoter. For combination *LbERF5*.1 & three binding site of *LbCCD4.1*’s promoter, we constructed a total of eight vectors to verify the binding specificity between *LbERF5.1* and the three binding sites of *LbCCD4.1*’s promoter (Fig. S9, see online supplementary material). All the plasmids were transformed into *Pichia Pastoris* GS115 and then the infective bacterial solution was injected into tobacco leaves. After cultivating 2 days, the injection site of the leaf tissue was used for protein extraction. The contents of firefly luciferase and renilla luciferase were determined using the Pierce™ Renilla-Firefly Dual Luciferase Assay Kit (Thermo Fisher Scientific, Waltham, MA, USA) and compared with the empty vector pGreenII 0800-LUC. The ratio fLUC/rLUC (firefly luciferase/renal luciferase) was used to measure the relative luciferase activity. The fluorescein was injected into tobacco leaves to determine the intensity of luciferase using a 4800 automatic chemiluminescence image analysis system (Tianneng, Shanghai, China).

### RNA-seq and qRT-PCR

The fruits of 14 *Lycium* accessions and the leaves of OE-*LbCCD4.1* and OE-*LbERF5.1* were sampled (three replicates) for total RNA isolation using RNAprep Pure Plant Kit (Tiangen Biotech, China). RNA-seq libraries were prepared and 150 bp paired-end sequences were performed in MetWare Co., Ltd (Wuhan, China). The clean data was obtained through the removal of reads that did not meet quality standards [84]. STAR package was used to map the clean reads to the reference genome of *Lycium* under default settings [85], followed by StringTie’s transcripts assembly and expression quantification using fragments per kilobase of transcript per million mapped reads (FPKM) method [86]. Differentially expressed genes (DEGs) were yielded by the following two criteria: (i): *P* value <0.01; and (ii): fold change ≥1.5 by applying DESeq [87]. The TFs of all the DEGs were annotated using PlantTFDB 5.0 (http://planttfdb.gao-lab.org/index.php). Finally, GO and KEGG enrichment analyses were performed independently based on DEGs from different group comparisons [88, 89].

The qRT-PCR primers of this study were designed using primer3 [82] (Table S6, see online supplementary material). Using BIO-RAD CFX Connect™ Amplification with *LbEf1a* as internal reference, the qRT-PCR was performed according to our previous report [90], which used the 2^−ΔΔCT^ method to convert the gene expression level [91].

## Acknowledgements

This work was sponsored by the National Natural Science Foundation of China (No. 32060359), the Key Research & Development Program of Ningxia Hui Autonomous Region (No. 2021BEF02002，2022BBF01001), the Innovative Research Group Project of Ningxia Hui Autonomous Region (No. 2021AAC01001) and the Innovation Team for Genetic Improvement of Economic Forests (No. 2022QCXTD04).

## Author contributions

J.Z. and W.A. conceived and designed the research. H.L., Y.Y., X. Zhang and X. Zhu. prepared the population material. H.L., L.D., X.L., T.H., and B.Z. performed sampling, sequencing, and transcriptome analyses. J.Z., Z.S., X.Q., and Y.C. contributed to the project discussion. J.Z., Y.X., and H.L. wrote the manuscript. J.Z., Y.X., and Y.C. revised the manuscript. All the authors read and approved the final manuscript.

## Data availability statement

Data supporting the findings of this work are available within the paper and the Supplementary Tables and Figures. The transcriptome clean sequencing data have been deposited into the National Center for Biotechnology Information Sequence Read Archive database (PRJNA936937).

## Conflict of interests 

All the authors declare that there is no conflict of interest.

## Supplementary data

Supplementary data is available at *Horticulture Research* online.

## Supplementary Material

Web_Material_uhad230
