# Supplementary material for: ERF5.1 modulates carotenoid accumulation by interacting with CCD4.1 in Lycium
Source: Hortic Res. 2023 Nov 17;10(12):uhad230. doi: 10.1093/hr/uhad230 (PMC10745278; doi:10.1093/hr/uhad230)
Supplement: Web_Material_uhad230 [file web_material_uhad230.zip › Figure S3.pdf]

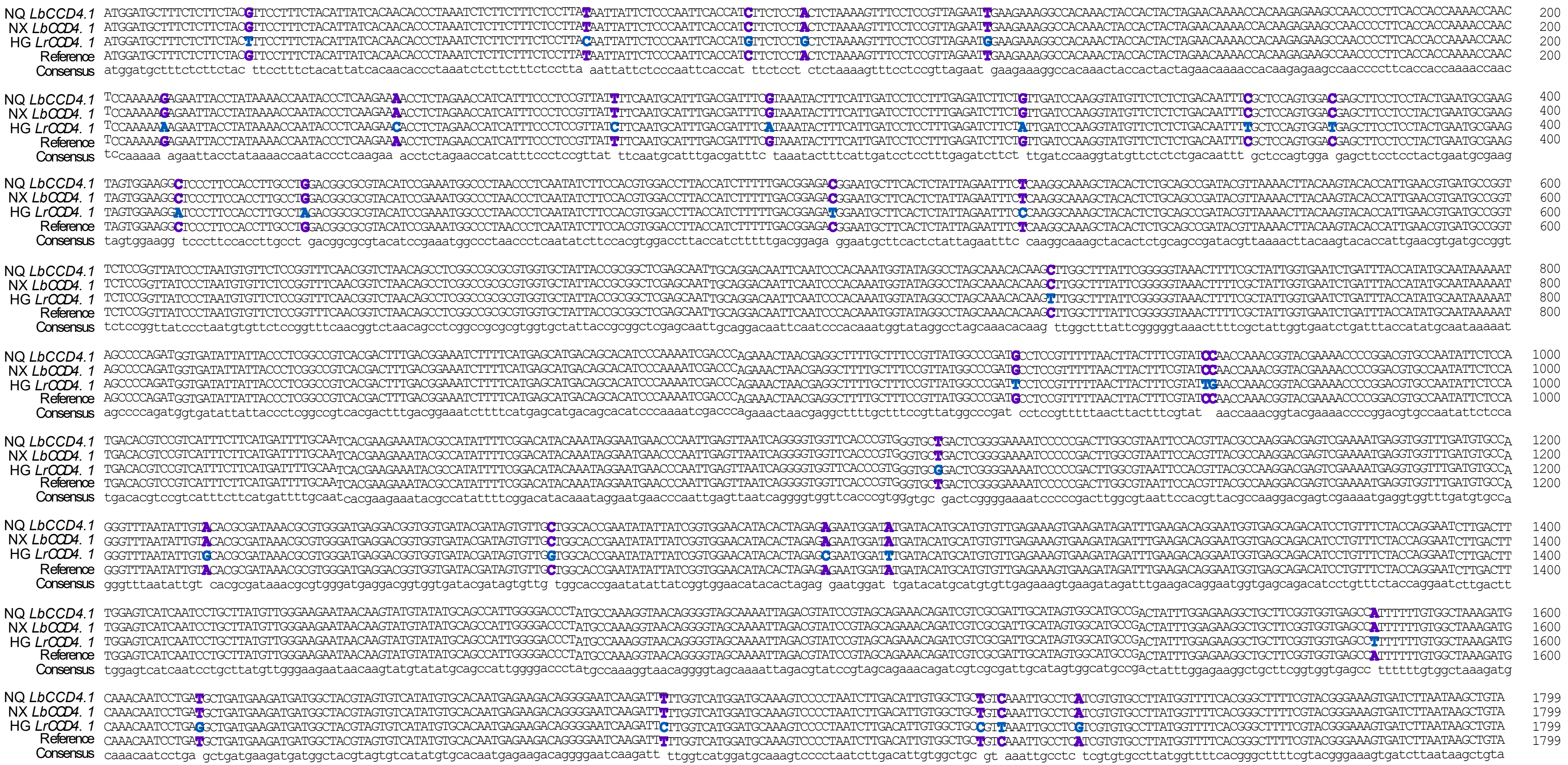
NQ LbCCD4.1
NX LbCCD4. 1
HG LrCCD4. 1
Reference
Consensus
200
400
600
800
1000
1200
1400
1600
1799
