# Supplementary figures and images for: ERF5.1 modulates carotenoid accumulation by interacting with CCD4.1 in Lycium
Source: Hortic Res. 2023 Nov 17;10(12):uhad230. doi: 10.1093/hr/uhad230 (PMC10745278; doi:10.1093/hr/uhad230)

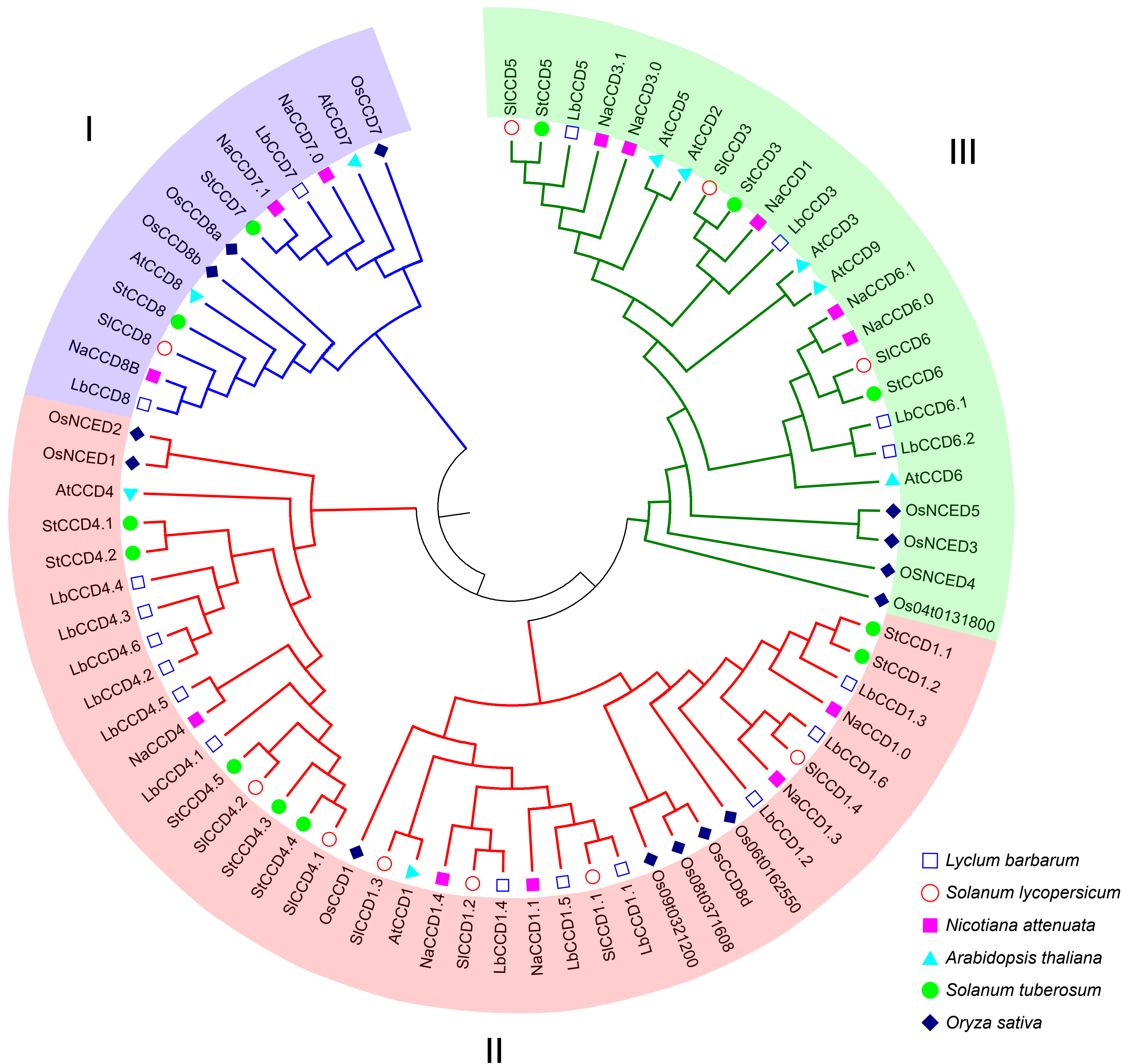

Supplement: Web_Material_uhad230 [file web_material_uhad230.zip › Figure S1.pdf]

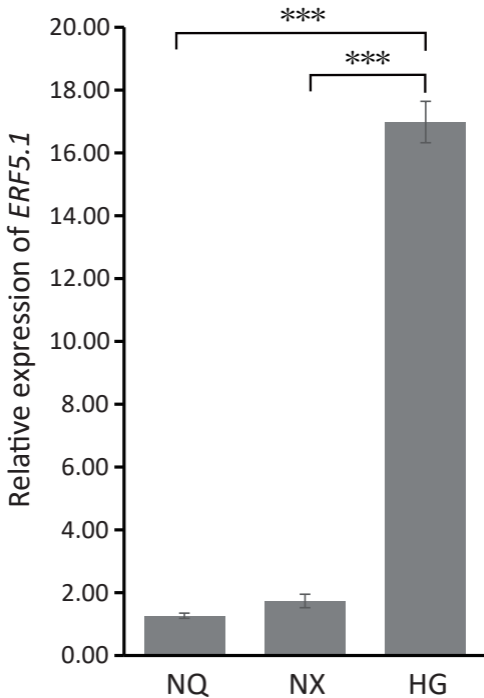

Supplement: Web_Material_uhad230 [file web_material_uhad230.zip › Figure S10.pdf]

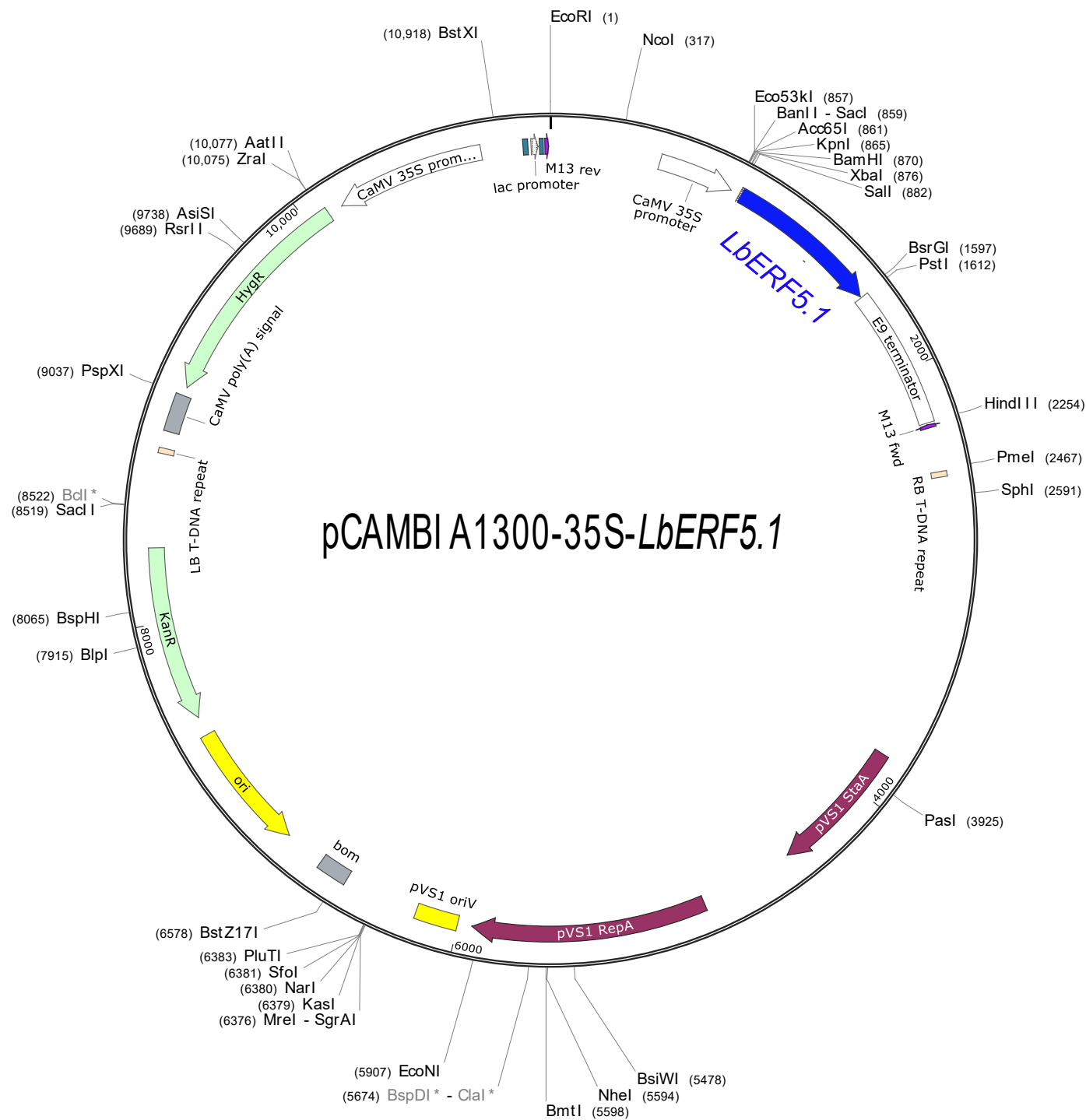

Supplement: Web_Material_uhad230 [file web_material_uhad230.zip › Figure S11.pdf]

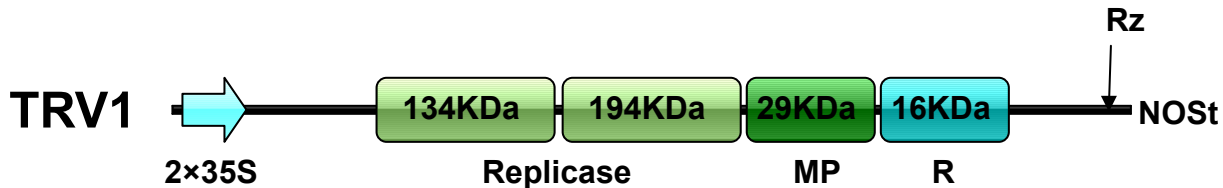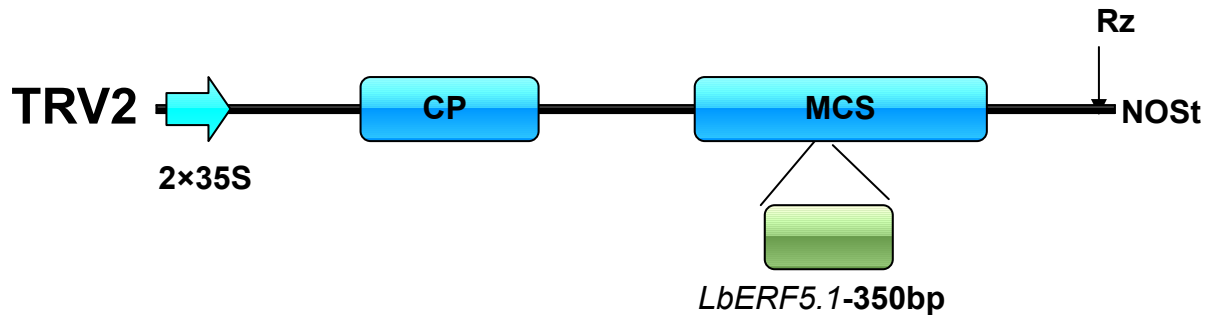

Supplement: Web_Material_uhad230 [file web_material_uhad230.zip › Figure S12.pdf]

DEGs of OE-*LbCCD4.1*

DEGs of OE-*LbERF5.1*

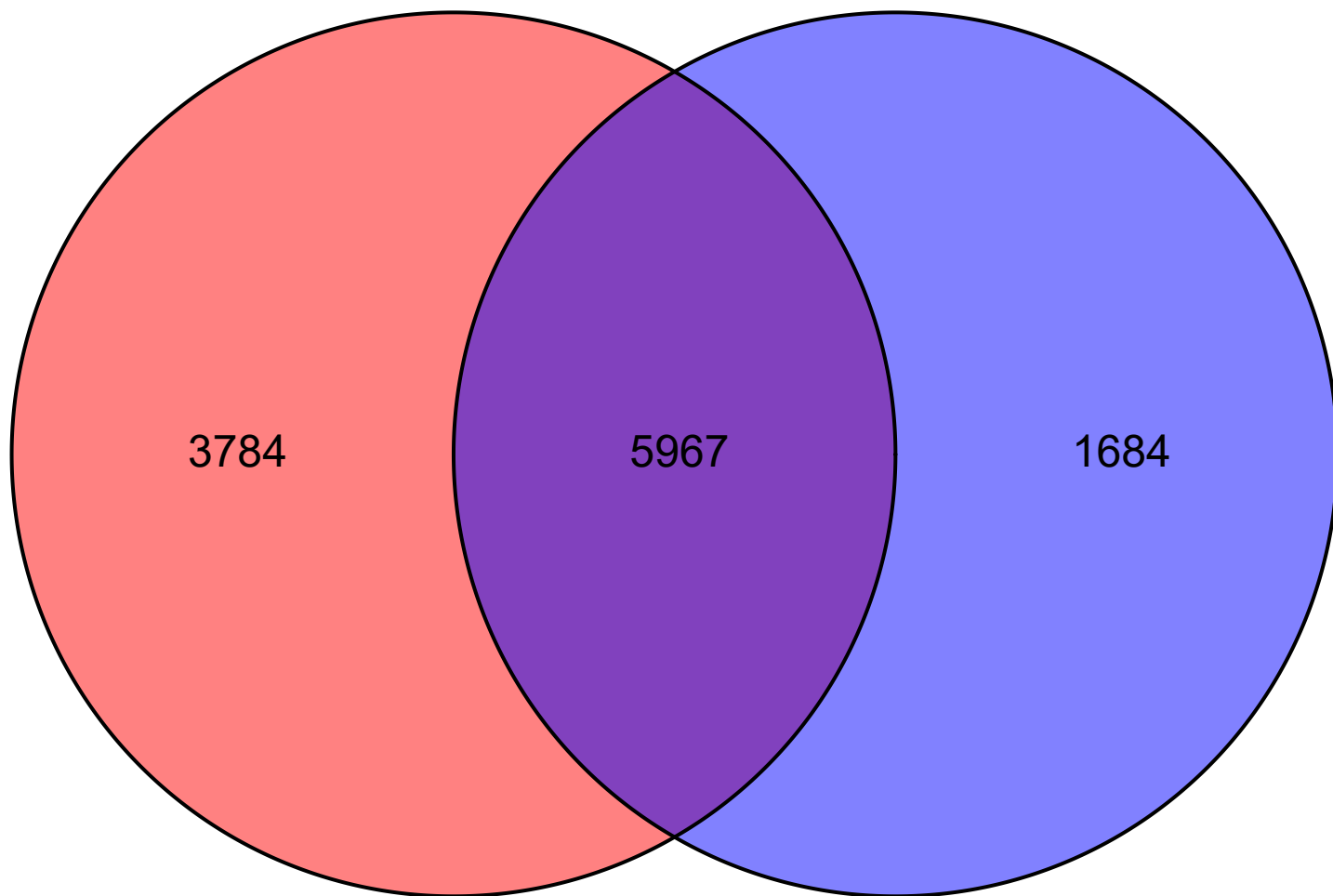

Supplement: Web_Material_uhad230 [file web_material_uhad230.zip › Figure S13.pdf]

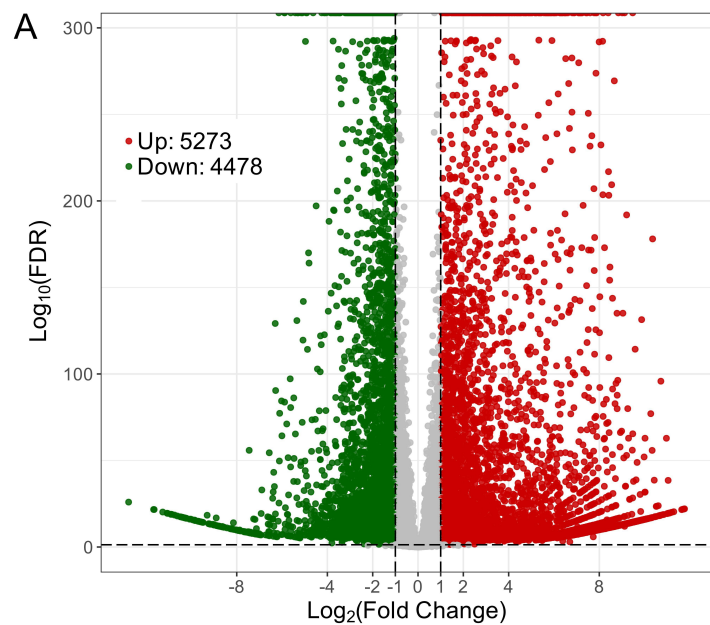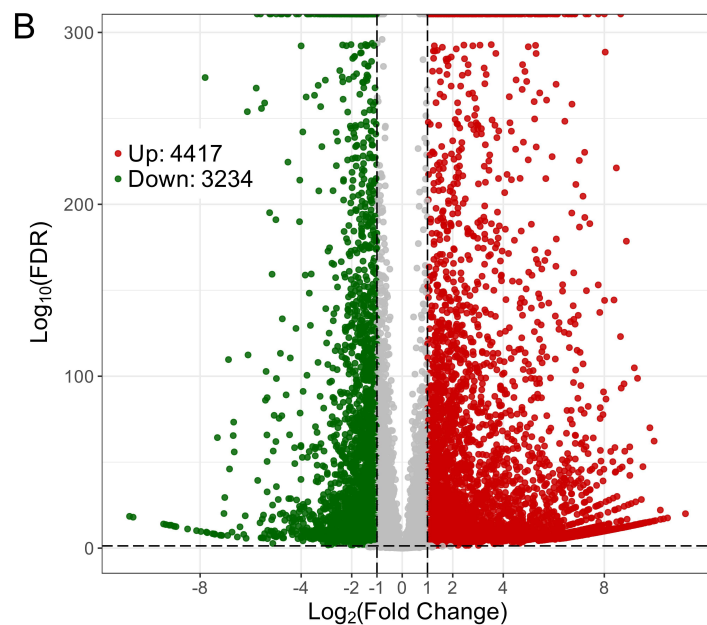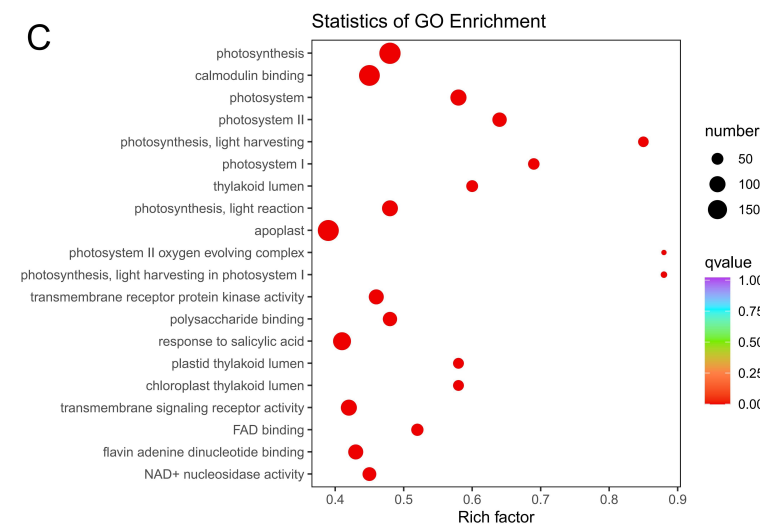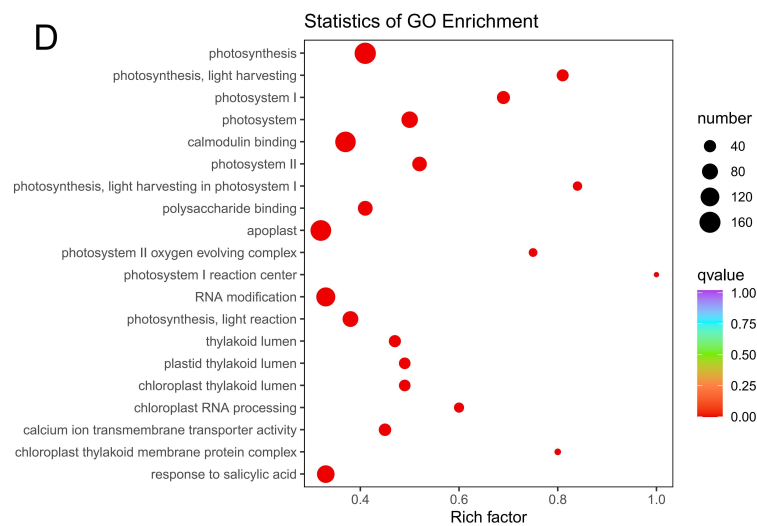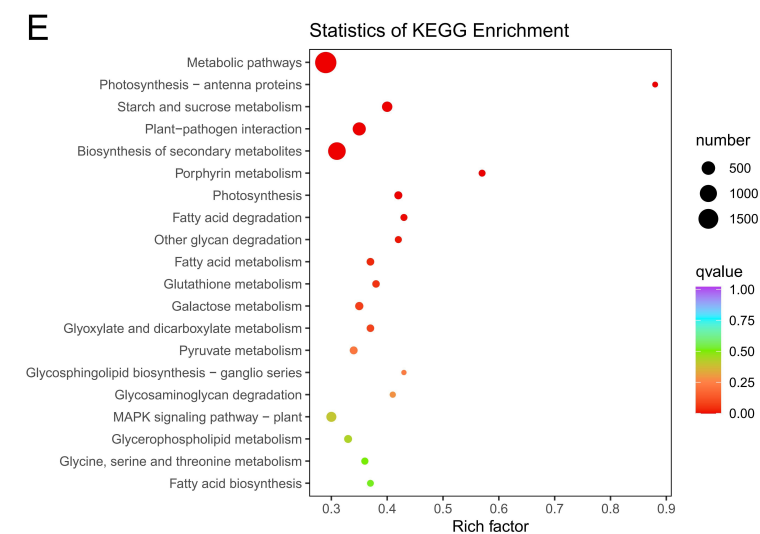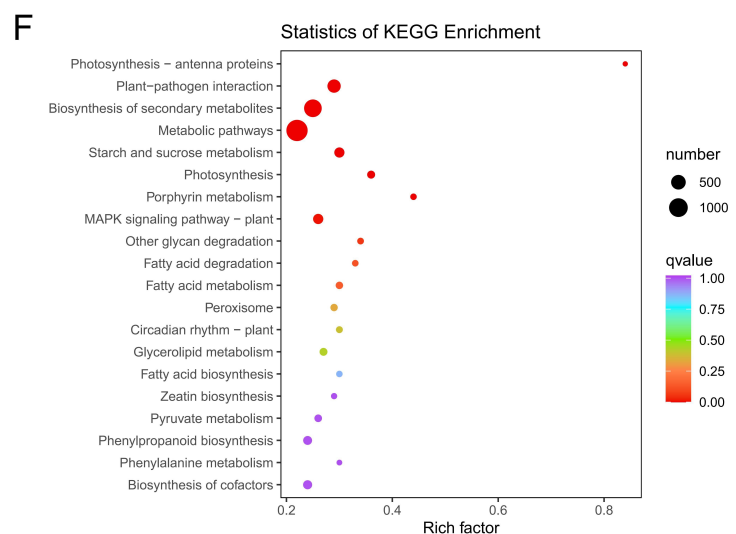

Supplement: Web_Material_uhad230 [file web_material_uhad230.zip › Figure S14.pdf]

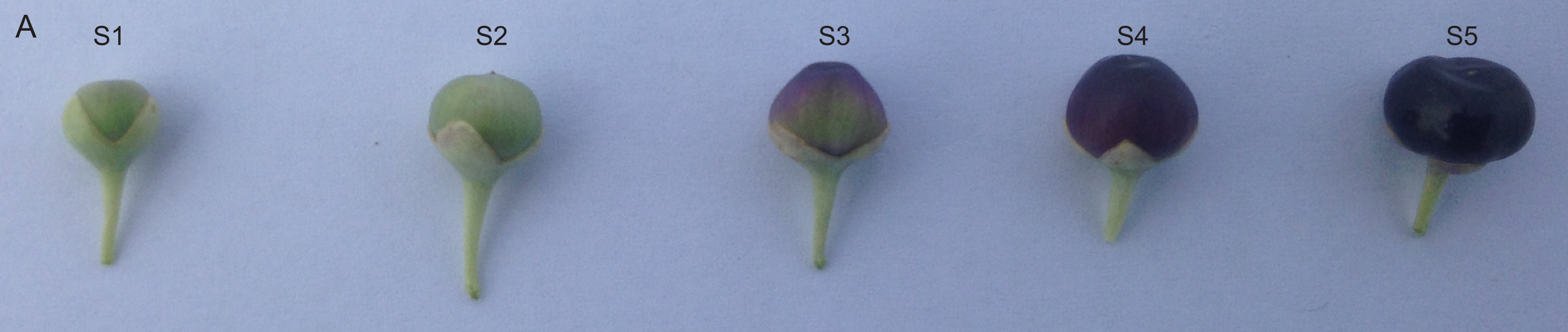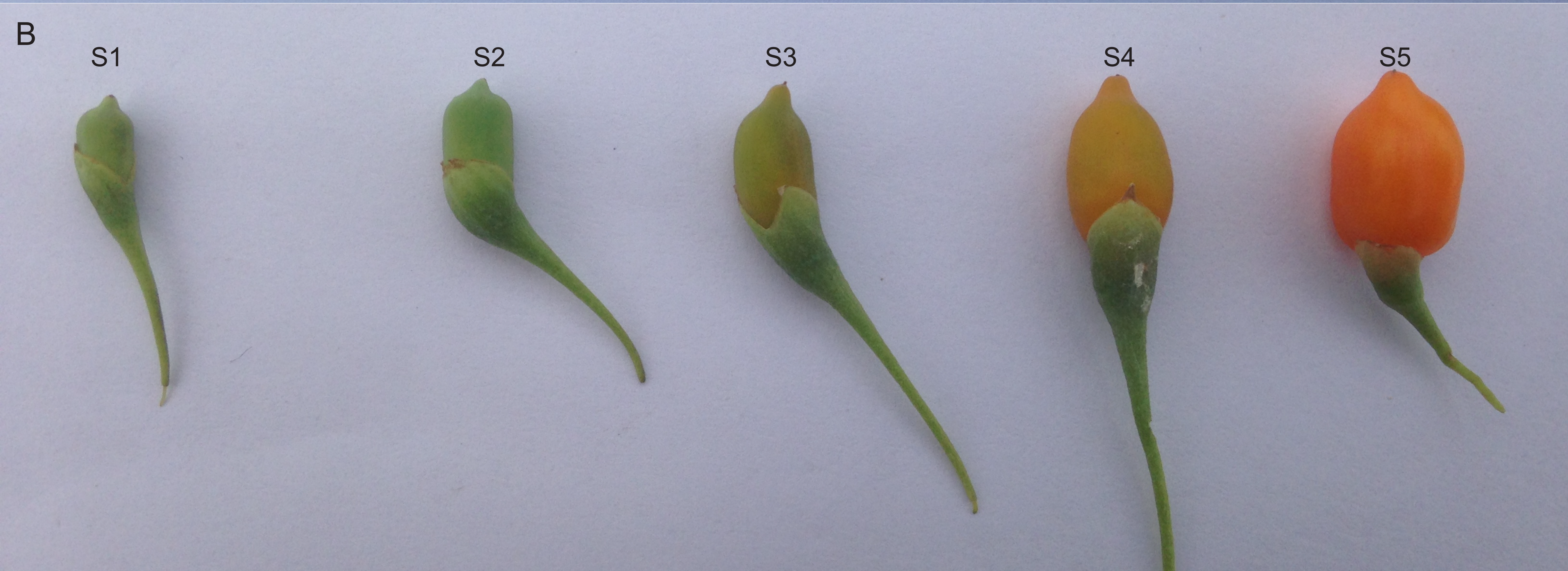

Supplement: Web_Material_uhad230 [file web_material_uhad230.zip › Figure S2.pdf]

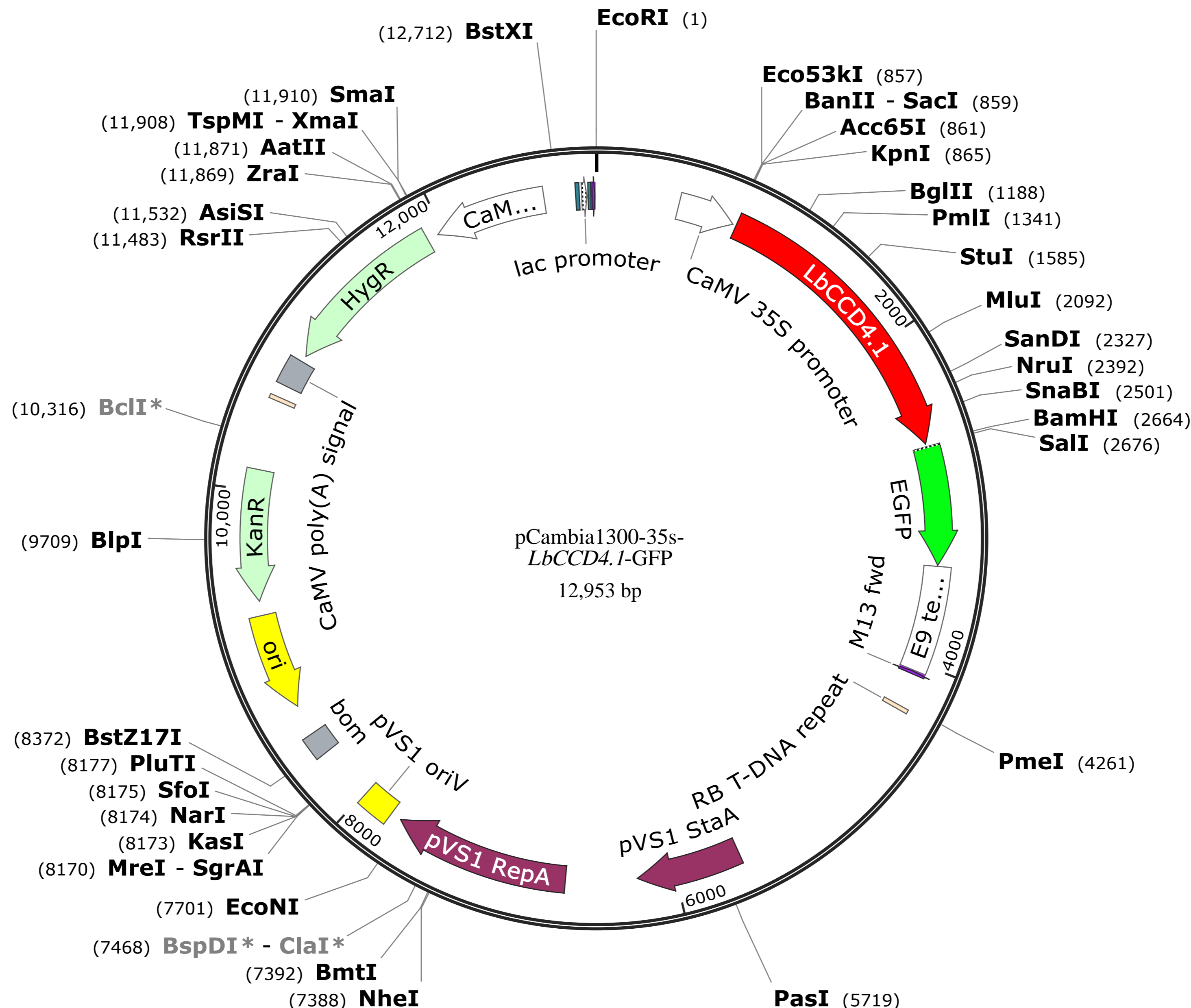

Supplement: Web_Material_uhad230 [file web_material_uhad230.zip › Figure S4.pdf]

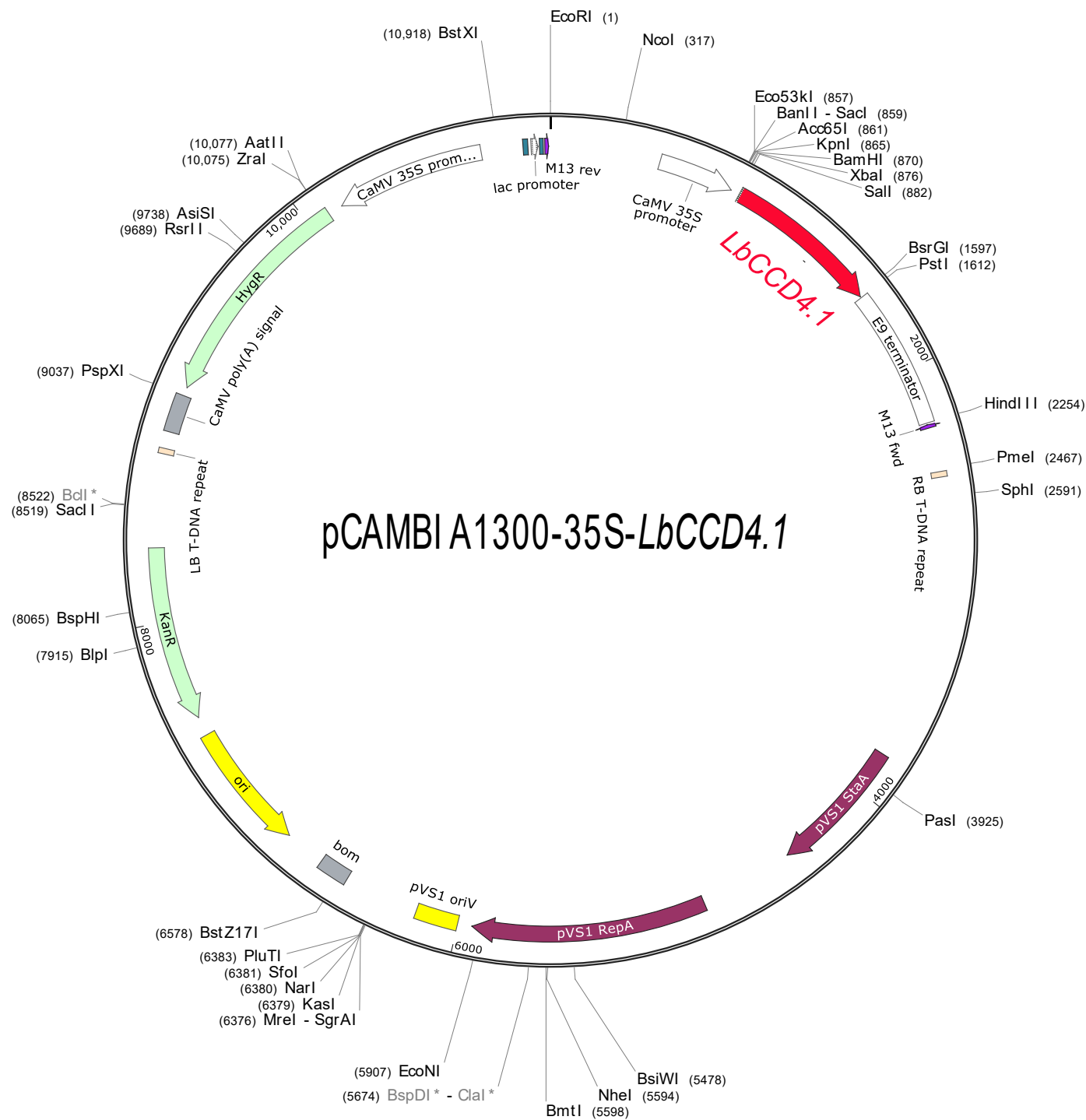

Supplement: Web_Material_uhad230 [file web_material_uhad230.zip › Figure S5.pdf]

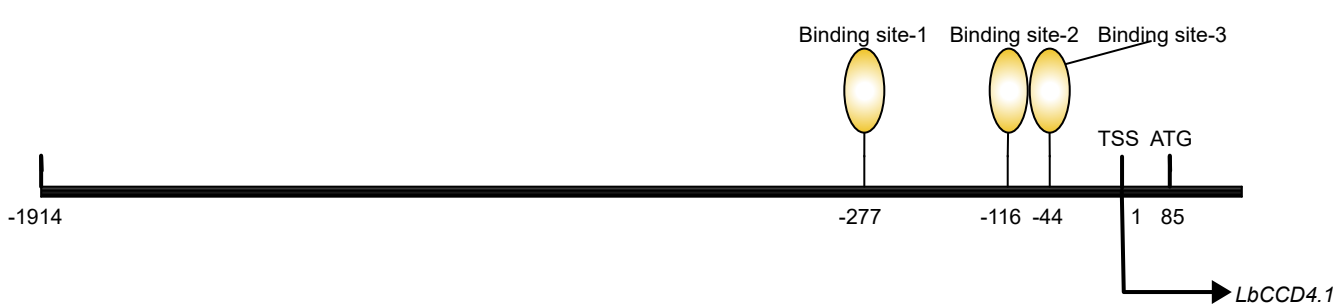

Supplement: Web_Material_uhad230 [file web_material_uhad230.zip › Figure S6.pdf]

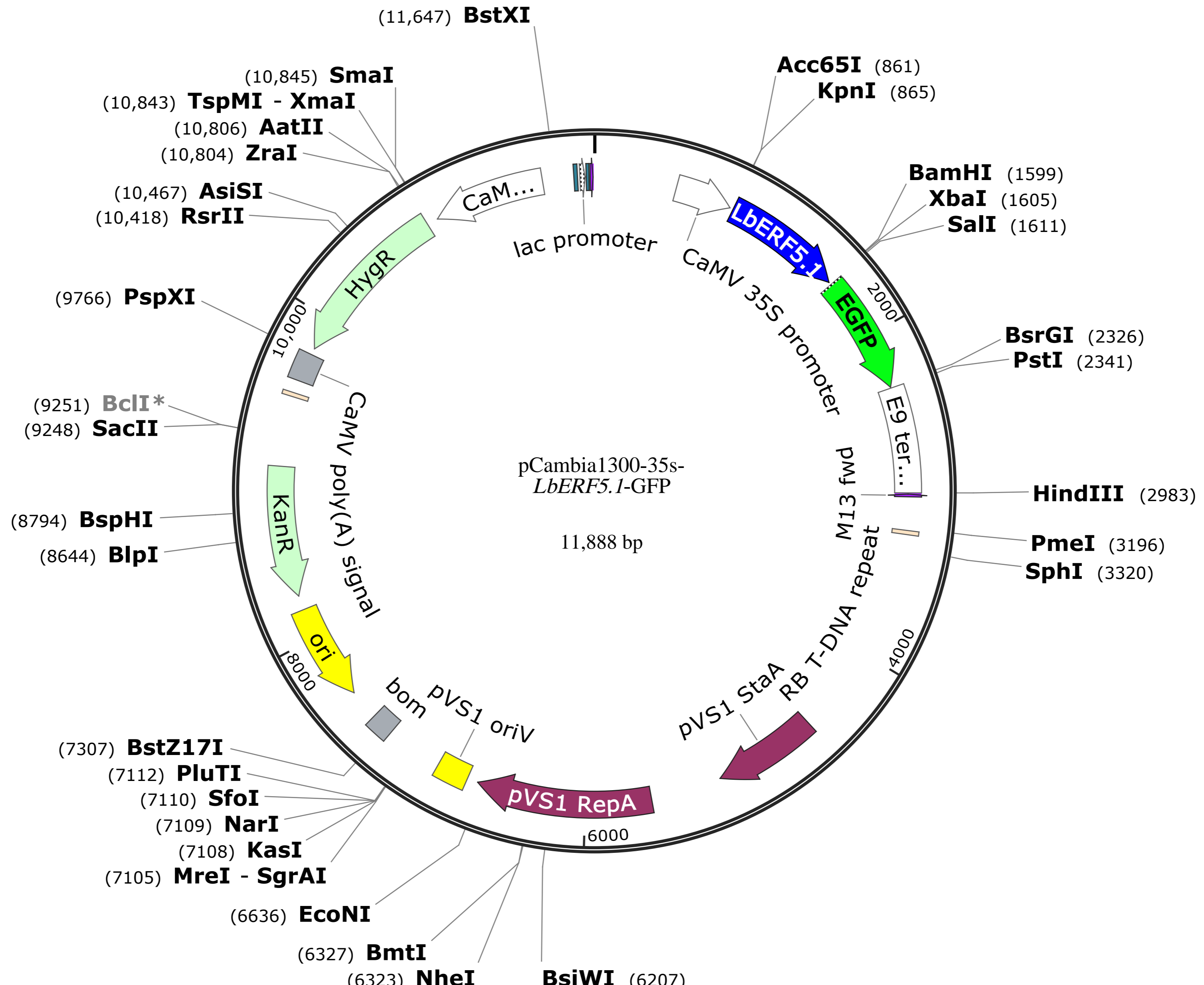

Supplement: Web_Material_uhad230 [file web_material_uhad230.zip › Figure S7.pdf]

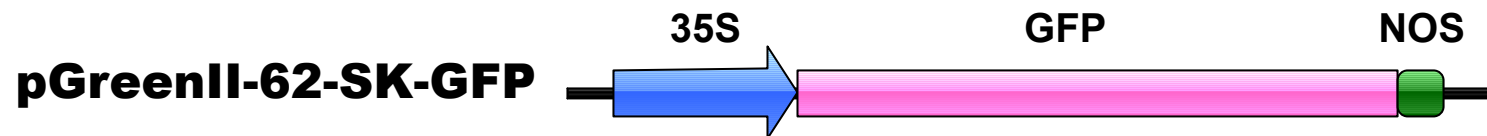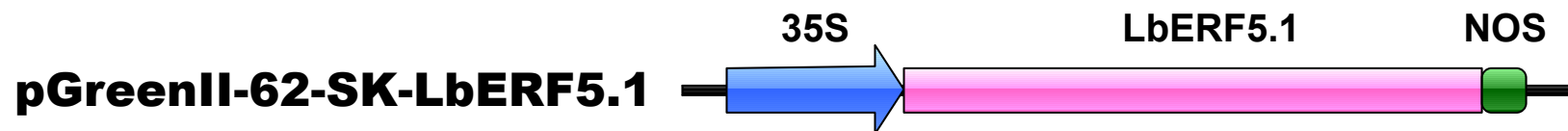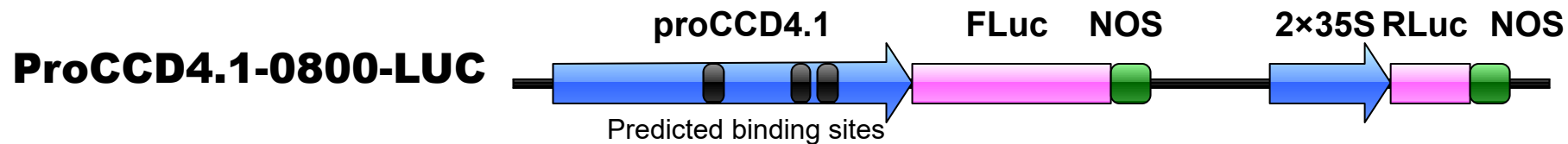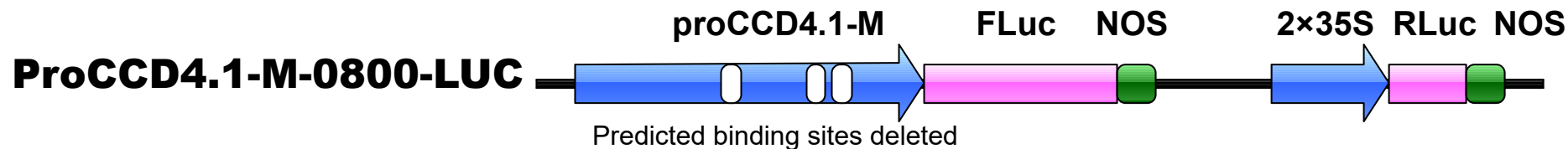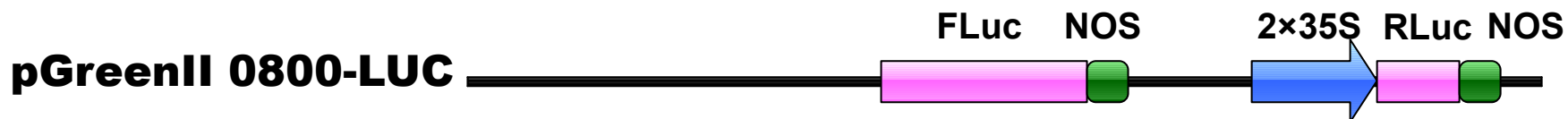

Supplement: Web_Material_uhad230 [file web_material_uhad230.zip › Figure S8.pdf]

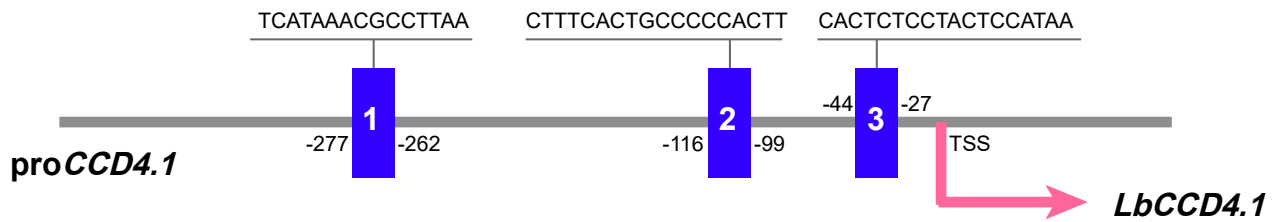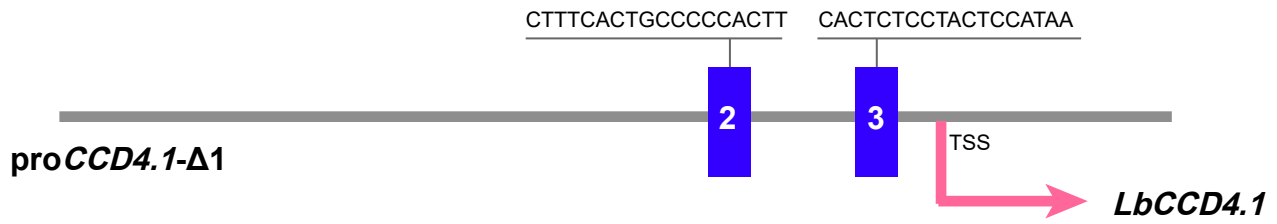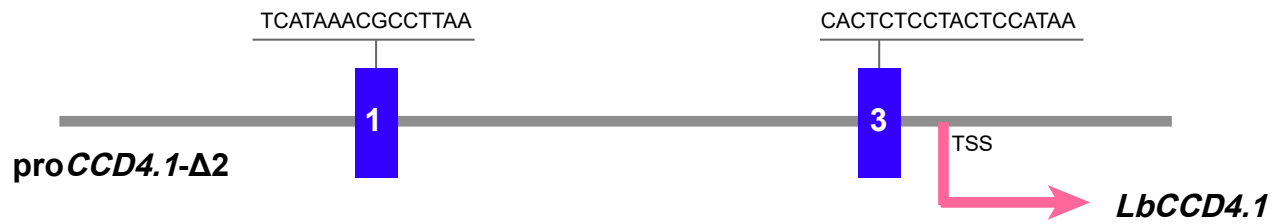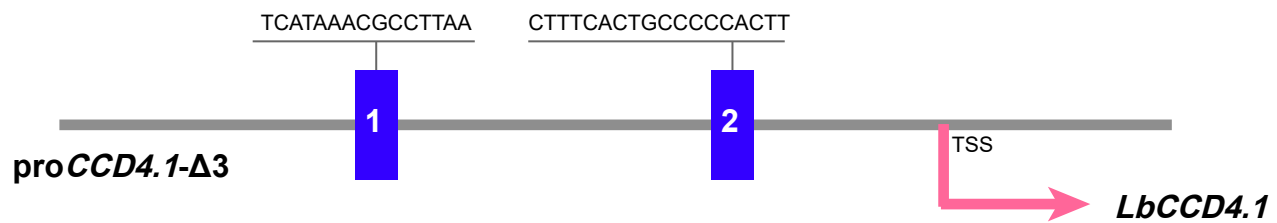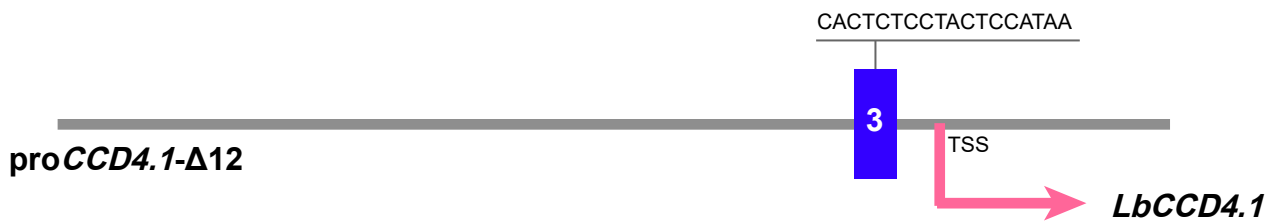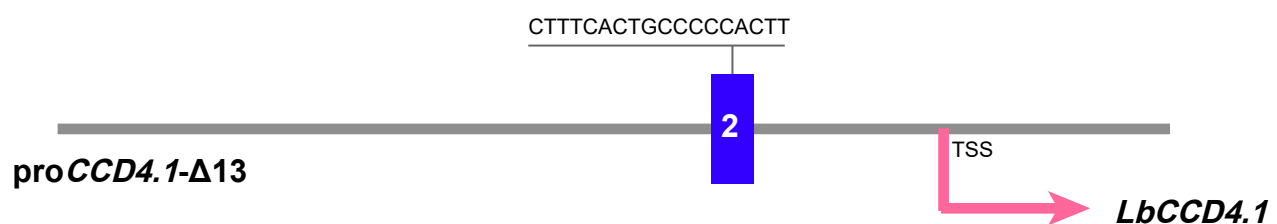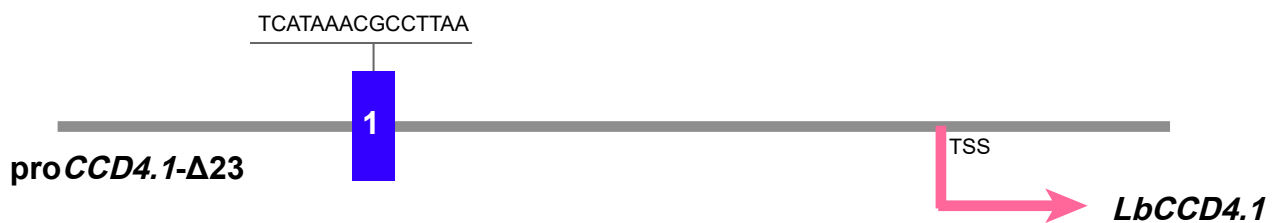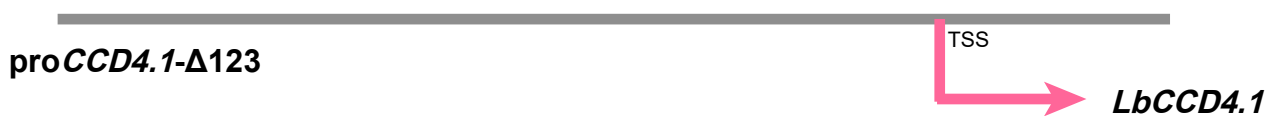

Supplement: Web_Material_uhad230 [file web_material_uhad230.zip › Figure S9.pdf]
